# Perfluoroalkyl substances (PFASs) as risk factors for breast cancer: a case–control study in Chinese population

**DOI:** 10.1186/s12940-022-00895-3

**Published:** 2022-09-09

**Authors:** Xuejun Li, Fengju Song, Xiaotu Liu, Anqi Shan, Yubei Huang, Zhengjun Yang, Haixin Li, Qiaoyun Yang, Yue Yu, Hong Zheng, Xu-Chen Cao, Da Chen, Ke-Xin Chen, Xi Chen, Nai-jun Tang

**Affiliations:** 1 Department of Occupational and Environmental Health, School of Public Health, Center for International Collaborative Research on Environment, Nutrition, and Public Health, Tianjin Key Laboratory of Environment, Nutrition, and Public Health, Tianjin Medical University, No.22 Qixiangtai Road, Heping District, Tianjin, 300070 China; 2 Department of Epidemiology and Biostatistics, Key Laboratory of Breast Cancer Prevention and Therapy in Ministry of Education, Key Laboratory of Molecular Cancer Epidemiology of Tianjin, National Clinical Research Center for Cancer, Tianjin Medical University Cancer Institute and Hospital, Tianjin, 300060 China; 3 School of Environment, Guangzhou Key Laboratory of Environmental Exposure and Health, and Guangdong Key Laboratory of Environmental Pollution and Health, Jinan University, Guangzhou, 510632 China; 4 The First Department of Breast Cancer, Tianjin Medical University Cancer Institute and Hospital, National Clinical Research Center for Cancer, Tianjin, 300060 China

**Keywords:** Perfluoroalkyl substances (PFASs), Breast cancer, Case–control study, Chinese population, Least absolute shrinkage and selection operator (LASSO), Bayesian kernel machine regression (BKMR), Quantile g-computation approach

## Abstract

**Background:**

Perfluoroalkyl substances (PFASs) are a large family of synthetic chemicals, some of which are mammary toxicants and endocrine disruptors. Recent studies have implicated exposure to PFASs as a risk factor for breast cancer in Europe and America. Little is known about the role of PFASs with respect to breast cancer in the Chinese population.

**Methods:**

Participants who were initially diagnosed with breast cancer at Tianjin Medical University Cancer Institute and Hospital between 2012 and 2016 were recruited as cases. The controls were randomly selected from the participants with available blood samples in the Chinese National Breast Cancer Screening Program (CNBCSP) cohort. Ultimately, we enrolled 373 breast cancer patients and 657 controls. Plasma PFASs were measured by an ultra-performance liquid chromatography (UPLC) system coupled to a 5500 Q-Trap triple quadrupole mass spectrometer. A logistic regression model with least absolute shrinkage and selection operator (LASSO) regularization was used to calculate odds ratios (ORs) and 95% confidence intervals (CIs) to assess the relationships between PFASs and breast cancer. The three most predictive variables in the LASSO model were selected from 17 PFASs, which was based on the optimal penalty coefficient (λ = 0.0218) identified with the minimum criterion. Additionally, Bayesian kernel machine regression (BKMR) and quantile g-computation models were applied to evaluate the associations between separate and mixed exposure to PFASs and breast cancer.

**Results:**

Perfluorooctanesulfonic acid (PFOS) exhibited the highest concentration in both the cases and controls. Perfluorooctanoic acid (PFOA) and perfluoro-n-decanoic acid (PFDA) were positively associated with breast cancer, and perfluoro-n-tridecanoic acid (PFTrDA) was negatively associated with breast cancer according to both the continuous-PFASs and the quartile-PFASs logistic regression models. Of note, PFOA was associated with the occurrence of estrogen receptor (ER)-, progesterone receptor (PR)-, and human epidermal growth factor receptor 2 (HER2)-positive breast cancer (OR_ER+_  = 1.47, 95% CI: 1.19, 1.80; OR_PR+_  = 1.36, 95% CI: 1.09, 1.69; OR_HER2_ = 1.62, 95% CI: 1.19, 2.21).

**Conclusions:**

Overall, we observed that PFASs were associated with breast cancer in Chinese women. Prospective cohort studies and mechanistic experiments are warranted to elucidate whether these associations are causal.

**Supplementary Information:**

The online version contains supplementary material available at 10.1186/s12940-022-00895-3.

## Background

Perfluoroalkyl substances (PFASs) are a group of fluorinated chemicals that have been used in consumer products and industrial applications for their stain-, grease-, and water-repellent properties since the 1950s. Most humans carry determinable burdens of PFASs. The extensive study by the U.S. National Health and Nutrition Examination Survey (NHANES) in 2013–2014 detected these compounds in the blood of over the 98% of the participants [1]. More than 90% of samples from the general population have detectable perfluorooctanoic acid (PFOA), perfluorooctanesulfonic acid (PFOS), and PFOS alternatives in China [2–4]. PFASs have attracted more concern and have been actively researched because of their extensive presence in the environment, persistent accumulation, and toxicity due to their long serum half-lives [5]. Of note, PFOS and PFOA have been added to the Stockholm Convention on persistent organic pollutants (POPs) [6]. Epidemiological and experimental studies have associated PFAS exposure with a series of toxicological effects that include changes in glucose and cholesterol, thyroid hormone alterations, reproductive and developmental toxicity, hepatotoxicity, and carcinogenic effects [7–9].

Breast cancer is the most common cancer among Chinese women. China accounts for 12.2% of new breast cancer diagnoses and 9.6% of all breast cancer deaths globally [10, 11]. In addition to genetic factors such as mutations in BRCA-1/2, risk factors for breast cancer include early menarche, late menopause, postmenopausal obesity, smoking, drinking alcohol, and high fat intake [12, 13]. Known risk factors can only explain less than one-third of the incidences, and lifestyle and environmental exposure can also alter the risk of breast cancer [14]. Environmental chemicals may play an important role in breast cancer. PFASs have been confirmed to exert weak xenoestrogen effects [7]. In addition, the International Agency for Research on Cancer (IARC) classified PFOA as a possible human carcinogen (Group 2B) in 2017, based on limited evidence that PFOA can cause testicular and renal cancer [15]. Many epidemiological studies have been implemented in Europe and the United States to assess breast cancer risks associated with PFAS exposure. Nonetheless, the literature is sparse, and the findings are controversial. Furthermore, little is known about the role of PFASs in the development of breast cancer in the Chinese population. Of note, several distinct characteristics have been observed in Chinese breast cancer patients, including a younger diagnostic age (45–55 years) compared to patients in Western countries and a pattern of risk factors only partially matching the known factors in high-income countries [10].

Given the limited research in this area and the biological plausibility of an association, we conducted a case–control study to evaluate the associations between individual and joint PFAS exposure and breast cancer by several statistical models.

## Methods

### Study design and participants

**Fig. 1:**
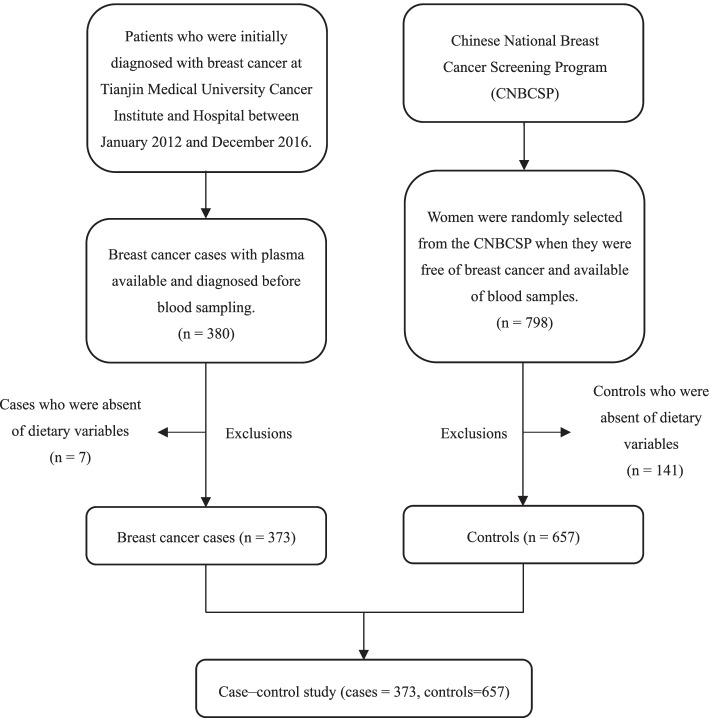
Flowchart of the case–control study

This study was approved by the Ethics Board of the Tianjin Medical University Cancer Institute and Hospital. Participants who were initially diagnosed with breast cancer by pathological examinations at Tianjin Medical University Cancer Institute and Hospital between January 2012 and December 2016 were recruited as cases. The blood samples of the patients were collected within a week after breast cancer diagnosis and before treatment. The controls included in this case–control study were randomly selected from the participants in the Chinese National Breast Cancer Screening Program (CNBCSP) cohort from a time period similar to that of the cases [16, 17]. We identified 380 cases and 798 controls with available blood samples. From these participants, those who had not completed the dietary questionnaire were excluded (cases = 7, controls = 141). In total, 373 cases and 657 controls were included in this study. Moreover, the clinical data, including the morphology, metastasis, staging, and hormone receptor status of breast tumors, were collected. The flowchart summarizing the inclusion process of the case–control study is presented in Fig. 1.

### Subtype classification

According to the standard for the molecular typing of breast cancer, tumors were identified as luminal A, luminal B, human epidermal growth factor receptor 2 (HER2), and basal-like subtypes [18]. Luminal A-like tumors were defined as estrogen receptor (ER)-positive, progesterone receptor (PR)-positive, HER2-negative, and low Ki67 expression. The remaining ER-positive tumors were classified as the luminal B subtype. HER2-driven tumors were categorized as ER-negative, PR-negative, and HER2 overexpression or amplification. Basal-like breast cancer was negatively expressed in ER, PR, and HER2 subtypes [19].

### Sample collection and analysis of PFASs in plasma

Blood samples were collected by licenced phlebotomists into 10 mL BD® tubes using standard phlebotomy techniques. Blood samples were collected from cases after diagnosis and before treatment. In the control group, the time of blood sample collection was the same as the screening time in the CNBCSP. The samples were stored at -80 °C until analysis. The detection methodology of PFASs has been previously described [2] and is detailed in the Supplemental Materials. The PFASs were quantified using an ultra-performance liquid chromatography (UPLC) system coupled to a 5500 Q-Trap triple quadrupole mass spectrometer (AB Sciex, Canada). PFASs include perfluorocarboxylated acids (PFCAs) and perfluorosulfonated acids (PFSAs). In summary, the levels of 38 PFASs were measured in this study, including five linear PFSAs (PFOS, PFBS, PFHxS, PFHpS, and PFDS), 11 linear PFCAs (PFOA, PFBA, PFPeA, PFHxA, PFHpA, PFNA, PFDA, PFUdA, PFDoA, PFTrDA, and PFTeDA), 17 PFOA and PFOS isomers (P1MHpS, P3MHpS, P3MHpA, P4MHpS, P4MHpA, P5MHpS, P5MHpA, P6MHpS, P6MHpA, P55DMHxS, P55DMHxA, P44DMHxS, P44DMHxA, P45DMHxS, P45DMHxA, P35DMHxS, and P35DMHxA), two chlorinated alternatives (11CL-PF3OUdS and 9CL-PF3ONS), and three fluorotelomer sulfonates (4:2FTS, 6:2FTS, and 8:2FTS). 13C8-PFOA was used as an internal standard, and nine isotopically labelled PFASs were applied as surrogate standards (Wellington Laboratories, Guelph, Ontario, Canada). The names, acronyms, and detection frequencies of the PFASs are presented in Table S1.

### Quality assurance and control

Quality assurance and control procedures included the analysis of matrix spiking samples, procedural blanks, and the monitoring of surrogate standard recoveries. The PFASs of interest were spiked into plasma and processed in five replicates along with two controls (only surrogate standards were spiked). The mean recoveries of individual PFASs ranged from 65 to 94% after subtracting the original values determined in the plasma. Two laboratory procedural blanks were processed in combination with every batch of 10 samples. All data were blank corrected prior to analysis. The recoveries of relevant surrogate standards in the samples ranged from 51 to 92%. Batch-specific blank correction was performed before data analysis. The recovery and standard deviation (SD) of each target compound are shown in Table S2.

### Statistical analysis

Concentrations below the limit of detection (LOD) were imputed as the detection limit divided by the square root of two. Normality was assessed using the Shapiro–Wilk test. The continuous variables were aggregated using the median and quartile range. Frequencies and percentages were applied for categorical variables. The Mann–Whitney-Wilcoxon test and Fisher’s exact test were used to make statistical comparisons of the demographic characteristics of the various groups. Spearman’s correlation was applied to describe pairwise relationships among the 17 PFASs (Figure S1). The level of PFASs was transformed using a natural logarithm (Ln) for a more normal distribution in the continuous model. Then, scatter plots of the residual distributions were used to detect the linearity of the continuous exposure variables, which is shown in Figure S2. Unconditional logistic regression models were applied to estimate odds ratios (ORs) and 95% confidence intervals (CIs) of breast cancer with respect to Ln (PFASs) concentrations. The ORs of PFAS concentrations that were analyzed as continuous variables with one SD increase in 17 Ln (PFASs, ng/ml) and quartile variables were both reported in this study by the logistic regression model. Variables were categorized according to the distribution of the PFAS concentrations in the controls, where the lowest quartile group was used as the reference group.

The least absolute shrinkage and selection operator (LASSO) regression model was used to minimize a large number of variables in the model and, importantly, had the unique feature of penalizing the absolute value of a regression coefficient [20, 21]. The regression coefficient of the regression model was based on the value of λ (λmin or λ1se). The larger the penalization is along with the greater the shrinkage of coefficients. When the estimates of weaker factors reached zero, the unnecessary variables were automatically removed, and only the strongest variables remained in the model. In this study, the most predictive PFAS variables were selected by λ (λ1se = 0.0218, Figure S3).

The Bayesian kernel machine regression (BKMR) model was used to evaluate the individual and combined effects of exposure to PFASs on breast cancer [22–24]. The 17 compounds were divided into three categories: PFCAs, PFSAs, and PFSA isomers. The ∑PFCAs included PFOA, PFNA, PFDA, PFUdA, PFDoA, PFTrDA, and PFTeDA. The ∑PFSAs included PFHxS, PFHpS, and PFOS. The ∑PFSA isomers included P3MHpS, P4MHpS, P5MHpS, P6MHpS, P45DMHxS, 11CL-PF3OUdS, and 9CL-PF3ONS. The three categories of PFAS mixtures were flexibly modelled and individual PFAS-breast cancer associations were estimated using a kernel function in BKMR. This method allows for potential nonlinear exposure–response functions. In addition, the variable posterior inclusion probabilities (PIPs) estimated by BKMR for each single PFAS represented the relative importance of the PFASs to the overall mixture effect. We ln-transformed and standardized PFAS concentrations in the BKMR model.

To further improve the robustness of the joint effects of PFAS mixtures calculated by BKMR, quantile g-computation approach was used. Quantile g-computation is a recently developed approach that combines the inferential simplicity of weighted quantile sum (WQS) regression with the flexibility of g-computation [25, 26]. In this study, quantile g-computation provided an estimate of the total effect of the three categories of PFAS mixtures on breast cancer and weights for the individual PFASs, which represented their relative contribution to the overall mixture effect. The weights represented the percentage of a single PFAS’s positive or negative relationship with breast cancer, with the weights in each direction adding up to 1.0.

The identification of confounders was performed according to the following criteria. First, variables were hypothesized to be risk factors for outcomes. Second, potential confounding variables identified by professional knowledge, traditional experience, and prior research were included in the adjusted models. Third, some important covariates were included in the adjusted models regardless of how they influenced the estimates [27–29]. The final multivariable models included terms for age at baseline (continuous), BMI (continuous), smoking (nonsmoker vs. current or former smoker), age at menarche (< 13 vs. ≥ 13 years), age at menopause (pre/peri-menopausal, < 51, or ≥ 51 years), parity (< 2 vs. ≥ 2), breastfeeding duration (< 13 vs. ≥ 13 months), use of estrogen or estrogen replacement therapy (no vs. yes), family history of breast cancer (no vs. yes), education level (< 13 vs. ≥ 13 years of education), income (< 1000, 1000–1999, 2000–2999, or ≥ 3000 Chinese RMB per month), and food consumption (rare, occasional, or often). Consumption of red meat, pickled, fried, smoked, and barbequed foods were separated into three levels: rare (defined as less than twice per week), occasional (defined as three or four times per week), and often (defined as more than five times per week). Alcohol consumption (nondrinker vs. current or former drinker) was not adjusted due to the small number of current and former drinkers (*n* = 5) in the case group. The LASSO, BKMR, and quantile g-computation models were adjusted for the same covariates.

All analyses were conducted using SPSS 26.0 and R version 4.2.0 (“glmnet”, “bkmr”, and “qgcomp” packages). A two-sided test and *P*-value < 0.05 were considered statistically significant.

## Results

The characteristics of the study participants are presented in Table 1. This study contained 373 cases and 657 controls. The median age was 50.6 years (IQR, 44.4–59.2 years) among the breast cancer patients and 53.0 years (IQR, 50.0–57.0 years) among the controls. Additionally, cases were more likely than controls to be current or former smokers (8.0% vs. 4.9%) and have a higher percentage (6.2% vs. 3.0%) of a family history of breast cancer. In the case group, the proportion of women who had more than two children and a longer breastfeeding duration (≥ 13 months) was higher than that in the control group. The distribution of estrogen replacement therapy use in breast cancer patients (4.1%) was similar to that in controls (3.4%) in the present study. There was no significant difference between the two groups in the level of education or monthly household income per capita (*p* > 0.05). The proportion of daily consumption of red meat, fried food, and smoked food did not differ between the two groups (*p* > 0.05). The frequencies of pickled and barbequed food intake were higher in the breast cancer patients than in the controls (*p* < 0.05).

**Table 1:** Demographic characteristics in cases and controls (*n* = 1030)

Characteristics	Case (*n* = 373)	Control (*n* = 657)	*P*-value
	**Median (IQR)/N (%)**	**Median (IQR)/N (%)**	
**Age at baseline (years)**	50.6 (44.4, 59.2)	53.0 (50.0, 57.0)	0.043
**BMI (kg/m**^**2**^**)**	24.4 (22.3, 26.5)	24.0 (22.1, 26.3)	0.150
**Smoking history**			0.043
Nonsmoker	343 (92.0%)	603 (95.1%)	
Current or Former smoker	30 (8.0%)	31 (4.9%)	
**Alcohol consumption**			0.072
Nondrinker	368 (98.7%)	611 (96.8%)	
Current or Former drinker	5 (1.3%)	20 (3.2%)	
**Age at menarche (years)**			< 0.001
< 13	28 (7.6%)	101 (15.5%)	
≥ 13	342 (92.4%)	551 (84.5%)	
**Age at menopause (years)**			< 0.001
Pre/Peri-menopausal	187 (51.2%)	184 (29.0%)	
< 51	109 (29.9%)	291 (46.0%)	
≥ 51	69 (18.9%)	158 (25.0%)	
**Parity**			< 0.001
< 2	246 (66.1%)	553 (85.3%)	
≥ 2	126 (33.9%)	95 (14.7%)	
**Breastfeeding duration (months)**			< 0.001
< 13	139 (39.7%)	422 (66.8%)	
≥ 13	211 (60.3%)	210 (33.2%)	
**Use of estrogen or estrogen replacement therapy**			0.598
No	355 (95.9%)	514 (96.6%)	
Yes	15 (4.1%)	18 (3.4%)	
**Family history of breast cancer**			0.015
No	349 (93.8%)	615 (97.0%)	
Yes	23 (6.2%)	19 (3.0%)	
**Education (years)**			0.200
< 13	284 (76.1%)	518 (79.6%)	
≥ 13	89 (23.9%)	133 (20.4%)	
**Monthly household income per capita (RMB/month)**			0.062
< 1000	39 (11.2%)	50 (7.7%)	
1000–1999	114 (32.9%)	187 (28.9%)	
2000–2999	117 (33.7%)	228 (35.3%)	
≥ 3000	77 (22.2%)	181 (28.0%)	
**Red meat consumption**			0.372
Rare	174 (46.6%)	324 (49.5%)	
Occasional	148 (39.7%)	235 (35.9%)	
Often	51 (13.7%)	95 (14.5%)	
**Pickled food consumption**			< 0.001
Rare	222 (59.5%)	597 (91.8%)	
Occasional	98 (26.3%)	41 (6.3%)	
Often	53 (14.2%)	12 (1.8%)	
**Fried food consumption**			0.169
Rare	309 (82.8%)	564 (86.1%)	
Occasional	50 (13.4%)	78 (11.9%)	
Often	14 (3.8%)	13 (2.0%)	
**Smoked food consumption**			0.103
Rare	352 (94.9%)	610 (96.7%)	
Occasional	15 (4.0%)	20 (3.2%)	
Often	4 (1.1%)	1 (0.1%)	
**Barbequed food consumption**			0.001
Rare	345 (92.5%)	616 (97.5%)	
Occasional	21 (5.6%)	14 (2.2%)	
Often	7 (1.9%)	2 (0.3%)	

Continuous variables were presented as median (25th, 75th); and categorical variables were presented as number (percentage)

*Abbreviations*: *BMI* Body mass index, *IQR* Interquartile range

A total of 38 PFASs were detected in this study, and the detection rates of 17 PFASs were greater than 80%. The concentrations of the 17 PFASs (ng/mL) in the plasma samples are shown in Table 2. The proportions above the LOD of the top seven PFASs in the control samples were 100% for PFOA, 100% for 9CL-PF3ONS, 99.90% for PFHxS, 99.61% for PFDA, 99.32% for 11CL-PF3OUdS, 99.22% for PFOS, and 99.22% for PFUdA. PFOS exhibited the highest concentration in both cases and controls, followed by 9CL-PF3ONS and PFOA. Cases had higher levels of PFOA and PFDA than controls.

**Table 2:** Distribution of the baseline plasma concentrations (ng/mL) of perfluoroalkyl substances (PFASs)

Compound	LOD (ng/ml)	Percent (> LOD)	Cases	Controls
			Median (IQR) (ng/mL)	Median (IQR) (ng/mL)
PFOA	0.116	100.00	3.72 (2.14, 6.30)	3.18 (2.27, 4.62)
PFNA	0.220	96.89	1.46 (0.83, 2.30)	1.76 (1.25, 2.34)
PFDA	0.010	99.61	1.04 (0.57, 1.71)	0.91 (0.64, 1.31)
PFUdA	0.009	99.22	0.68 (0.42, 1.03)	0.98 (0.58, 1.60)
PFDoA	0.010	96.80	0.07 (0.04, 0.11)	0.10 (0.06, 0.15)
PFTrDA	0.028	89.71	0.09 (0.02, 0.25)	0.52 (0.36, 0.82)
PFTeDA	0.008	80.97	0.01 (0.01, 0.04)	0.05 (0.03, 0.08)
PFHxS	0.012	99.90	1.18 (0.45, 2.24)	1.59 (0.81, 2.47)
PFHpS	0.030	97.67	0.45 (0.20, 0.80)	0.54 (0.27, 0.94)
PFOS	0.969	99.22	11.14 (6.36, 17.39)	12.64 (8.23, 18.06)
P3MHpS	0.043	92.43	0.86 (0.44, 1.51)	0.87 (0.47, 1.56)
P4MHpS	0.211	91.65	1.11 (0.55, 1.96)	1.20 (0.61, 2.14)
P5MHpS	0.120	96.31	1.79 (0.82, 2.93)	1.85 (0.94, 3.17)
P6MHpS	0.076	84.27	1.61 (0.75, 2.82)	1.77 (0.84, 3.04)
P45DMHxS	0.006	84.08	0.08 (0.02, 0.14)	0.09 (0.04, 0.15)
11CL-PF3OUdS	0.001	99.32	0.11 (0.06, 0.21)	0.11 (0.06, 0.21)
9CL-PF3ONS	0.007	100.00	8.63 (4.49, 15.04)	9.37 (4.85, 15.20)

*Abbreviations*: *LOD* Limit of detection, *IQR* Interquartile range, *PFOA* Perfluorooctanoic acid, *PFNA* Perfluoro-n-nonanoic acid, *PFDA* Perfluoro-n-decanoic acid, *PFUdA* Perfluoro-n-undecanoic acid, *PFDoA* Perfluoro-n-dodecanoic acid, *PFTrDA* Perfluoro-n-tridecanoic acid, *PFTeDA* Perfluoro-n-tetradecanoic acid, *PFHxS* Perfluorohexane sulfonate, *PFHpS* Perfluoroheptane sulfonate, *PFOS*, Perfluorooctanesulfonic acid, *P3MHpS* Perfluoro-3-methylheptane sulfonate, *P4MHpS* Perfluoro-4-methylheptane sulfonate, *P5MHpS* Perfluoro-5-methylheptane sulfonate, *P6MHpS* Perfluoro-6-methylheptane sulfonate, *P45DMHxS* Perfluoro-4,5-dimethylhexane sulfonate, *11CL-PF3OUdS* Potassium 11-chloroeicosafluoro-3-oxaundecane-1-sulfonate, 9CL-PF3ONS Potassium 9-chlorohexadeca-fluoro-3-oxanonane-1-sulfonate

**Fig. 2:**
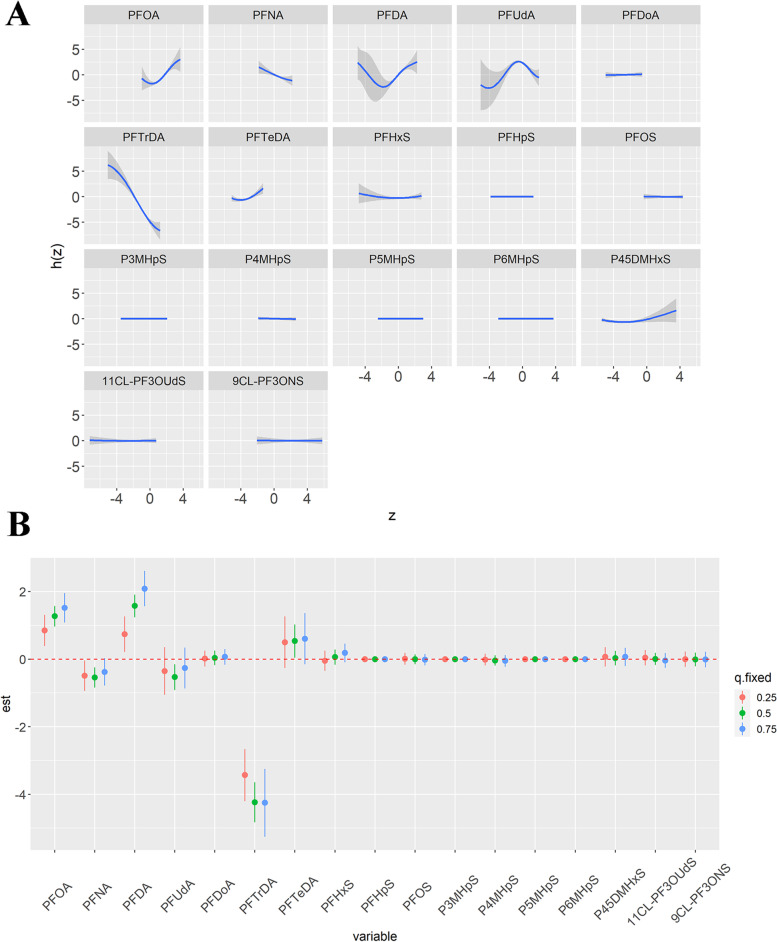
Univariate exposure–response functions between exposure to the 17 PFASs and breast cancer calculated by the Bayesian kernel machine regression (BKMR) model. (**A**) Plot of univariate exposure response cross section; (**B**) Plot of the effects of single PFAS on breast cancer. Differences in the association of a single PFAS with breast cancer at the 75th and 25th percentiles when the remaining 16 variables were fixed at the 25th, 50th, and 75th percentiles

As shown in Figure S1, the Spearman’s rank correlations among 17 PFASs were calculated. As several of the PFASs were highly correlated, the LASSO regression model was used to reduce data dimensions and determine the most powerful PFAS variables before logistic regression model was performed. The crude and adjusted ORs of breast cancer for the 17 PFASs as continuous and quantile variables in a logistic regression model without LASSO regularization are shown in Table S3. We further evaluated the effects of the PFASs as continuous variables using LASSO regression followed by logistic regression. The LASSO logistic model minimized multicollinearity among the 17 PFASs and assessed the relationship between the selected PFASs and breast cancer. We used a LASSO model to build a PFASs classifier (Figure S3) and selected the most powerful PFAS variables (λ = 0.0218, *n* = 3). Finally, the logistic regression model was used to evaluate the associations between the selected variables (PFOA, PFDA, and PFTrDA) and breast cancer. The results showed that PFOA (OR = 3.32, 95% CI: 2.32, 4.75) and PFDA (OR = 2.22, 95% CI: 1.55, 3.17) were positively associated with breast cancer (Table 3). We also found that an increase in the SD in Ln (PFTrDA) was negatively associated with breast cancer (OR = 0.03, 95% CI: 0.02, 0.06; Table 3). The results from logistic regression with LASSO-selected PFASs showed that both the positive and negative associations were enhanced (OR_PFOA_, 3.32 vs. 1.57; OR_PFDA_, 2.22 vs. 1.27; OR_PFTrDA_, 0.03 vs. 0.10) compared to the results from single-pollutant logistic regression. To further improve the robustness of the results of the selected PFASs calculated by the LASSO regression model, BKMR was used to estimate individual PFAS-breast cancer associations (Fig. 2). Among the 17 PFASs, the substances that had the highest conditional PIPs were PFOA (1.00), PFDA (1.00), PFUdA (1.00), and PFTrDA (1.00) (Table S4). Regarding the association between individual PFAS and breast cancer, while setting all other exposure variables at their median values, positive and significant associations were observed between PFOA and PFDA and breast cancer (Fig. 2A, B). Of note, a negative association existed between PFTrDA and breast cancer (Fig. 2A, B). Compared with the ORs shown in Table 3, the results calculated by BKMR were consistent with those calculated by LASSO regression, revealing the robustness of the associations between the PFASs and breast cancer.

**Table 3:** Crude and adjusted odds ratios (ORs) for the risk of breast cancer relative to the natural logarithm transformed concentrations of three selected perfluoroalkyl substances (PFASs) in a logistic regression model

(ng/ml)	Cases/ controls	Crude OR (95%CI)	Cases/ controls	Adjusted OR (95%CI)
PFOA	373/657	2.39 (1.88, 3.05)*	315/447	3.32 (2.32, 4.75)*
PFDA	373/657	2.00 (1.54, 2.58)*	315/447	2.22 (1.55, 3.17)*
PFTrDA	373/657	0.04 (0.03, 0.06)*	315/447	0.03 (0.02, 0.06)*

Final multivariable model were adjusted for age at baseline (years), BMI (kg/m^2^), smoking history, age at menarche (years), age of menopause (years), parity, breastfeeding duration (months), use of estrogen or estrogen replacement therapy, family history of breast cancer, education (years), monthly household income per capita (RMB/month), red meat consumption, pickled, fried, smoked, and barbecued food consumption. The OR and 95% CI of breast cancer were estimated by one SD increase in ln-transformed PFOA, PFDA, and PFTrDA as continuous variables

*Abbreviations*: *OR* Odds ratio, *CI* Confidence interval, *PFOA* Perfluoro-n-octanoic acid, *PFDA* Perfluoro-n-decanoic acid, *PFTrDA* Perfluoro-n-tridecanoic acid

^*^ *p*-value for trend < 0.05

In addition to exploring the associations between individual PFAS exposure and breast cancer, the relationships between the three categories of PFASs including ∑PFCAs (PFOA, PFNA, PFDA, PFUdA, PFDoA, PFTrDA, and PFTeDA), ∑PFSAs (PFHxS, PFHpS, and PFOS), and ∑PFSA isomers (P3MHpS, P4MHpS, P5MHpS, P6MHpS, P45DMHxS, 11CL-PF3OUdS, and 9CL-PF3ONS), and breast cancer were also investigated in this study based on the BKMR and quantile g-computation models. As shown in Figure S4 and Figure S5, the results calculated by the two models were consistent. According to the results calculated by the quantile g-computation model, ∑PFCAs (OR = 0.86, 95% CI: 0.83, 0.88) and ∑PFSAs (OR = 0.92, 95% CI: 0.89, 0.95) were negatively associated with breast cancer. Exposure to PFSA isomers had no significant association with breast cancer (OR = 0.97, 95% CI: 0.94, 1.01).

Based on the variables selected by the LASSO model (PFOA and PFDA), the logistic regression model was further used to evaluate the associations between the plasma level of one SD increase in Ln (PFOA, ng/ml) and Ln (PFDA, ng/ml) and breast cancer stratified by subtypes or receptor expression status (Table 4). Interestingly, we observed that PFOA exposure was more likely to be associated with ER-positive (OR = 1.47, 95% CI: 1.19, 1.80) and PR-positive (OR = 1.36, 95% CI: 1.09, 1.69) breast cancer. Additionally, exposure to PFOA also showed positive associations with the luminal A (OR = 1.36, 95% CI: 1.08, 1.71) and HER2 (OR = 1.62, 95% CI: 1.19, 2.21) subtypes of breast cancer. We found that PFDA was positively related to ER-positive (OR = 1.19, 95% CI: 1.01, 1.41) breast cancer only in the crude model.

**Table 4:** Multinomial logistic regression models for the natural logarithm transformed PFOA and PFDA concentrations and breast cancer risk according to different subtypes of breast cancer

(ng/ml)	N	**PFOA**		**PFDA**	
		Crude OR (95% CI)	Adjusted OR (95% CI)	Crude OR (95% CI)	Adjusted OR (95% CI)
Control	657	Ref	Ref	Ref	Ref
ER-	96	1.01 (0.81, 1.26)	1.08 (0.82, 1.41)	0.93 (0.76, 1.14)	0.94 (0.72, 1.22)
ER +	218	1.43 (1.22, 1.67)*	1.47 (1.19, 1.80)*	1.19 (1.01, 1.41)*	1.21 (0.98, 1.50)
PR-	131	1.00 (0.82, 1.21)	1.03 (0.81, 1.30)	1.01 (0.84, 1.22)	0.93 (0.73, 1.18)
PR +	183	1.42 (1.19, 1.68)*	1.36 (1.09, 1.69)*	1.06 (0.90, 1.26)	1.04 (0.83, 1.30)
Luminal A	168	1.23 (1.03, 1.46)*	1.36 (1.08, 1.71)*	1.12 (0.94, 1.34)	1.12 (0.89, 1.42)
Luminal B	52	1.03 (0.77, 1.38)	1.06 (0.74, 1.50)	0.84 (0.66, 1.07)	0.83 (0.61, 1.13)
HER2	65	1.46 (1.13, 1.90)*	1.62 (1.19, 2.21)*	1.28 (0.97, 1.70)	1.26 (0.91, 1.76)
Basal	29	1.72 (1.20, 2.47)*	1.45 (0.93, 2.24)	1.20 (0.80, 1.80)	1.06 (0.70, 1.60)

Note: According to the standard for molecular typing of breast cancer, cases were also identified as luminal A subtype, luminal B subtype, HER2 subtype, and basal-like subtypes. Final multivariable models adjusted for age at baseline (years), BMI (kg/m^2^), smoking history, age at menarche (years), age of menopause (years), parity, breastfeeding duration (months), use of estrogen or estrogen replacement therapy, family history of breast cancer, education (years), monthly household income per capita (RMB/month), red meat consumption, pickled, fried, smoked, and barbecued food consumption. The OR and 95% CI of breast cancer were estimated by one SD increase in ln-transformed PFOA and PFDA as continuous variables

*Abbreviations*: *OR* Odds ratios; *CI* Confidence interval, *PFOA* Perfluorooctanoic acid, *PFDA* Perfluoro-n-decanoic acid, *ER* Estrogen receptor, *PR* Progesterone receptor, *HER2* Human epidermal growth factor receptor 2

^*^ *p*-value for trend < 0.05

## Discussion

The case–control study demonstrated that plasma concentrations of PFOA and PFDA were positively associated with breast cancer, and PFTrDA was negatively associated with breast cancer in Chinese women. PFOA was more likely to be associated with the ER-, PR-, and HER2-positive breast cancer. The associations between joint and individual exposure to PFASs and breast cancer were examined by LASSO regression, BKMR, and quantile g-computation models, which proved the robustness of the conclusions.

The study population was exposed to a variety of perfluorinated compounds. PFOS, 9CL-PF3ONS, and PFOA were the top three perfluorinated compounds with the highest serum concentrations. The median serum concentrations (data not shown) measured in the study population were 12.18 ng/mL for PFOS, 3.35 ng/mL for PFOA, 0.94 ng/mL for PFDA, and 0.38 ng/ml for PFTrDA; these fit within the range of exposure levels reported in the C8 Health Project, a cross-sectional study conducted with 408 women in Shenyang, China, in which mean PFAS concentrations of 19.56 ng/ml for PFOS, 6.41 ng/ml for PFOA, 0.94 ng/ml for PFDA, and 0.68 ng/ml for PFTrDA were found [30].

This case–control study supported and extended the epidemiological findings on PFAS-breast cancer associations. PFOA and PFDA were positively associated with breast cancer based on this study. Two prospective studies [26, 31] both reported a positive association between PFOA and breast cancer, which was consistent with our findings. Moreover, a case–control study from the Greater Manila Area [32] found positive associations between breast cancer and the 3rd [OR (95% CI) = 4.09 (1.03, 21.08)] and 4th quantile groups [OR (95% CI) = 9.26 (2.54, 45.10)] of PFDA concentrations compared to the reference group. Furthermore, negative associations between PFOS and PFTrDA and breast cancer were observed both in our research and in a cross-sectional study conducted in Japan (OR_PFOS_ = 0.15, 95% CI: 0.06, 0.39; OR_PFTrDA_ = 0.14, 95% CI: 0.05, 0.35) [33]. In contrast to our results, no significant association of PFOA with breast cancer was observed in case–control study with an Inuit population [34] and the California Teachers Study [35]. The plasma concentration of PFOA in the Inuit population study [34] fit within the range of exposure levels of our population, and the inconsistencies may be explained by different genetic backgrounds and susceptibility to environmental contaminants. It should be noted that the main difference between our study population and the California population [35] was the time of blood sampling, which was performed after the cases received treatment. Therefore, the effect of cancer treatment on internal PFAS levels could not be eliminated. The discrepancies found in these epidemiological studies may reflect the different exposure profiles and susceptibility of the study population, as well as the characteristics of the study design. Notably, the positive association between PFOS and breast cancer observed in the Inuit population study may be partly explained by the high PFOS exposure. There are suspicions that several other POPs, which are present in Inuit people at considerably greater levels, may also have an impact on breast cancer. The risk of breast cancer in Inuit women may be increased by exposure to other carcinogenic or heterogeneous substances, such as PCBs [36].

Another interesting finding of this study is that PFOA is more likely to be associated with ER-, PR-, and HER2-positive breast cancer. In the French E3N cohort, low levels of PFOS and PFOA were related to receptor-negative tumors, which highlights the need to consider PFAS exposure as a potential risk factor for breast cancer [31]. No association was found between the PFOA serum concentration and ER-positive breast cancer in a case–control study of Taiwanese women either in the young (age ≤ 50 years, OR = 1.41, 95% CI: 0.77, 2.56) or older groups (age > 50 years, OR = 0.70, 95% CI: 0.35, 1.42) [37]. The inconsistency may be explained by the lower PFOA concentrations (1.77 ng/mL) reported in the Taiwanese study compared to those reported in our study (3.35 ng/mL). Future epidemiological studies are warranted to validate the current conclusions.

Furthermore, the negative associations between PFCAs and PFSAs and breast cancer were observed in our study. The mechanisms of a possible negative association are not well understood. Nevertheless, some suggestive evidence has been reported. This study indicated that PFTrDA may contribute most to the negative association between PFCAs and breast cancer based on the results calculated by quantile g-computation models. We speculated that the negative relationship may be partly explained by the change in thyroid function induced by PFTrDA exposure. T4 is a proliferative factor in vitro for breast cancer cells [38]. A previous study showed that the elimination of the clinical action of endogenous T4 in breast cancer patients has favorably affected the course of cancer [39]. Of note, Ji et al. revealed that the concentration of PFTrDA was negatively correlated with total T4 in Korean females [40]. Our study also revealed that PFHxS contributed most to the negative association between PFSAs and breast cancer. Alterations in lipid metabolism induced by PFHxS may be involved in the negative relationship. LDL could stimulate the migration of MDA-MB-231 cells [41]. The overexpression of the STARD3 gene, which encodes a cholesterol-binding membrane protein, could decrease cell adhesiveness, promote breast cancer metastases, and be associated with a poor prognosis for breast cancer patients [42]. Nevertheless, previous studies have reported significant negative associations of PFHxS with TC, and non-HDL [43, 44]. Moreover, differences between the results of single and mixed PFASs exposure may be partly related to statistical models. Individual components may act differently in the presence of additional chemicals, making the overall evaluation of mixtures in the environment difficult [45]. To be prudent, the results for PFAS must be considered with caution.

Stratified analyses of parity and breastfeeding duration were conducted in our study. We found that multi-parity women had a lower risk of breast cancer when exposed to PFOA or PFTrDA than nulliparous or single-birth women (Table S5). Women who had breastfed for a longer period of time (≥ 13 months) had a decreased risk of breast cancer than those who breastfed for a shorter amount of time due to PFOA or PFTrDA exposure (Table S6). The findings partly supported the hypothesis that parity and breastfeeding can transfer PFASs [46] and are associated with lower serum PFAS concentrations [47, 48], which may reduce the risk of breast cancer. Nonetheless, we observed that the multi-parity participants or women who had breastfed for more than 13 months were more sensitive to PFDA exposure than those with fewer children or shorter periods of breastfeeding.

The potential mechanisms underlying PFAS-breast cancer relationships involve four aspects: (1) PFASs in vitro can affect hormone receptors, disrupt endocrine homeostasis, and increase the risk of adverse health effects [49–51]; (2) PFASs can activate the peroxisome proliferator activated receptor alpha (PPARα) and induce oxidative stress [52–54]; (3) PFASs can induce epigenetic alterations, such as DNA methylation and histone modifications, which are implicated in tumorigenesis [55]; and (4) PFASs can cause reproductive toxicity, which can delay mammary gland development and increase susceptibility to carcinogens in offspring [56, 57].

Our study has some specific strengths. First, the associations between individual and joint exposure to PFASs and breast cancer were examined by several statistical models, and the consistency reflected the robustness of the conclusions. Second, this research benefited from a large number of cases that could be further grouped by hormone receptors status, and ultimately identified the breast cancer subtypes that are more sensitive to PFAS exposure, which can provide evidence for mechanistic studies. Several limitations should be noted in this study. First, the temporal relationships between PFAS exposure and breast cancer could not be ascertained based on the cross-sectional design of the research. Second, one major route of exposure to PFASs is through the ingestion of contaminated food or water [58]. The dietary variables included in this study may be insufficient, and data for some food categories, such as seafood, takeout food, and packaged food, which may be associated with PFASs [59, 60], have not been collected. Third, only one measurement of PFASs concentrations at the time of diagnosis among the study population was obtained, which may not accurately reflect the individuals' whole exposure scenario. However, it can be assumed that the general population may be exposed to a constant level of PFASs due to the slowly changing environmental PFASs concentrations, which were caused by the PFASs’ resistance to hydrolysis, photolysis, and microbial degradation because of the high-energy carbon–fluorine bond [61], and a long half-life in the human body [5]. To corroborate our findings, longitudinal follow-up studies from adolescence onwards are needed, and further exploration is necessary to determine whether any mediators cause or influence the observed relationships between PFAS levels and breast cancer.

## Conclusions

In conclusion, we found that the plasma concentrations of PFOA and PFDA were positively associated with breast cancer, and PFTrDA was negatively associated with breast cancer in Chinese women. PFOA was more likely to be associated with ER-, PR-, and HER2-positive breast cancer. These associations warrant attention in prospective cohort studies and well-designed toxicological experiments to elucidate whether these relationships are causal.

## Supplementary Information

**Additional file 1. **

## Data Availability

The datasets used and/or analyzed during the current study are available from the corresponding author on reasonable request.

## Abbreviations

PFASs: Perfluoroalkyl substances
NHANES: National Health and Nutrition Examination Survey
PFOA: Perfluorooctanoic acid
PFOS: Perfluorooctanesulfonic acid
POPs: Persistent organic pollutants
IARC: The International Agency for Research on Cancer
ER: Estrogen receptor
CNBCSP: Chinese National Breast Cancer Screening Program
HER2: Human epidermal growth factor receptor 2
PR: Progesterone receptor
UPLC: Ultra-performance liquid chromatography
PFCAs: Perfluorocarboxylated acids
PFSAs: Perfluorosulfonated acids
PFNA: Perfluoro-n-nonanoic acid
PFDA: Perfluoro-n-decanoic acid
PFUdA: Perfluoro-n-undecanoic acid
PFDoA: Perfluoro-n-dodecanoic acid
PFTrDA: Perfluoro-n-tridecanoic acid
PFTeDA: Perfluoro-n-tetradecanoic acid
PFHxS: Sodium perfluoro-1-hexanesulfonate
PFHpS: Sodium perfluoro-1-heptanesulfonate
P3MHpS: Perfluoro-3-methylheptane sulfonate
P4MHpS: Perfluoro-4-methylheptane sulfonate
P5MHpS: Perfluoro-5-methylheptane sulfonate
P6MHpS: Perfluoro-6-methylheptane sulfonate
P45DMHxS: Perfluoro-4,5-dimethylhexane sulfonate
11CL-PF3OUdS: Potassium 11-chloroeicosafluoro-3-oxaundecane-1-sulfonate
9CL-PF3ONS: Potassium 9-chlorohexadeca-fluoro-3-oxanonane-1-sulfonate
SD: Standard deviation
LOD: Limit of detection
Ln: Natural logarithm
OR: Odds ratio
CI: Confidence interval
LASSO: Least absolute shrinkage and selection operator
BKMR: Bayesian kernel machine regression
PIPs: Posterior inclusion probabilities
WQS: Weighted quantile sum
PPAR: Peroxisome proliferator activated receptor
