# Supplementary material for: Perfluoroalkyl substances (PFASs) as risk factors for breast cancer: a case–control study in Chinese population
Source: Environ Health. 2022 Sep 9;21:83. doi: 10.1186/s12940-022-00895-3 (PMC9463854; doi:10.1186/s12940-022-00895-3)
Supplement: Supplementary file 1 — Additional file 1. [file 12940_2022_895_MOESM1_ESM.docx]

***Part 1***

***Analysis of PFAS in Plasma***

Approximately 100 μL of each plasma sample was spiked with isotopically labeled standards and vortexed. After the sample was loaded onto a Captiva EMR-Lipid cartridge (1 mL, Agilent Technologies), 4 mL of acetonitrile containing 0.1% formic acid (v/v) was added to the cartridge and allowed to stand for 5 min. Target analytes were eluted under a low vacuum of 2–4 psi. The extraction procedure was conducted on the Biotage Extrahera automation system (Biotage, Sweden). The extract was concentrated and then filtered through a 0.2 μm nylon filter (VWR, Radnor, PA). The final extract contained 100 μL of a mixture of methanol and water (1:1, v/v). The PFASs were quantified using an ultra-performance liquid chromatography (UPLC) system coupled to a 5500 Q-Trap triple quadrupole mass spectrometer (AB Sciex, Canada). Chromatographic separation was performed on an ACQUITY UPLC BEH shield RP18 column (2.1 mm × 100 mm, 1.7 μm particle size; Waters).

The mobile phase consisted of phase A of 5 mM ammonium formate in water (pH = 4) and phase B of methanol. A flow rate of 300 μL/min was used. The gradient was programmed as: 0-2 min, 40% B; 2-3 min, ramped to 66% B (linear); 3-12 min, 66% ramped to 70% B (linear), followed by a linear increase to 100% B in 2 min (held for 2 min) and then a change to 40% B in 0.1 min (held for 6 min). The MS was equipped with a TurboIonSpray® electrospray ionization (ESI) probe operated in the multiple reaction monitoring (MRM) mode. The curtain gas (CUR), ion source 1 (Gas 1), and ion source 2 (Gas 2) were 30 psi, 30 psi, and 40 psi, respectively. The temperature of the ESI source was 400°C.

***Part 2***

***Quality Assurance and Control***

Quality assurance and control procedures included the analysis of matrix spiking samples, procedural blanks, and monitoring of surrogate standard recoveries. The PFASs of interest were spiked into plasma (Guangzhou Future Biotechnology, China) and processed in five replicates along with two controls (only surrogate standards were spiked) (Liu et al. 2020). The mean recoveries of individual PFASs ranged from 65% to 94% after subtracting the original values determined in the plasma. Two laboratory procedural blanks were processed in combination with every batch of 10 samples. All data were blank-corrected prior to analysis. The recoveries of relevant surrogate standards in the cohort samples ranged from 51% to 92%. Batch specific blank correction was performed before data analysis. The recovery and SD of each target compounds should be listed in Table S2. Reported concentrations were corrected using the recoveries of relevant surrogate standards. The limit of quantification (LOQ) of an analyte, defined as its response at 10-fold the standard deviation of the noise, ranged from 0.02 to 0.36 ng/mL.

***Part 3***

***Supplemental Tables (Table S1-Table S6)***

**Table S1.** Names, acronyms, and detection frequency of target perfluorinated chemicals.

| Acronym | Chemical name | Quantification transitions | Surrogate standards | %detected | LOQ (ng/ml) |
| --- | --- | --- | --- | --- | --- |
| PFBA | Perfluoro-n-butanoic acid | 213 > 169/69 | MPFBA | 0.763% | 0.795 |
| PFPeA | Perfluoro-n-pentanoic acid | 263 > 219/69 | MPFBA | 0.000% | 0.519 |
| PFHpA | Perfluoro-n-heptanoic acid | 363 > 169/319 | MPFHxA | 2.034% | 0.060 |
| PFOA | Perfluoro-n-octanoic acid | 413 > 369/169 | MPFOA | 100.000% | 0.116 |
| PFNA | Perfluoro-n-nonanoic acid | 463 > 169/419 | MPFNA | 97.119% | 0.220 |
| PFDA | Perfluoro-n-decanoic acid | 513 > 469/219 | MPFDA | 99.661% | 0.010 |
| PFUdA | Perfluoro-n-undecanoic acid | 563 > 519/269 | MPFUdA | 99.068% | 0.009 |
| PFDoA | Perfluoro-n-dodecanoic acid | 613 > 569/269 | MPFUdA | 96.864% | 0.010 |
| PFTrDA | Perfluoro-n-tridecanoic acid | 663 > 619/269 | MPFOA | 93.644% | 0.028 |
| PFTeDA | Perfluoro-n-tetradecanoic acid | 713 > 669/369 | MPFOA | 80.763% | 0.008 |
| PFBS | Potassium perfluoro-1-butanesulfonate | 299 > 80/99 | MPFHxS | 40.424% | 0.010 |
| PFHxS | Sodium perfluoro-1-hexanesulfonate | 399 > 80/99 | MPFHxS | 100.000% | 0.012 |
| PFHpS | Sodium perfluoro-1-heptanesulfonate | 449 > 80/99 | MPFHxS | 98.305% | 0.030 |
| PFOS | Sodium perfluoro-1-octanesulfonate | 499 > 80/99 | MPFOS | 99.322% | 0.969 |
| PFDS | Sodium perfluoro-1-decanesulfonate | 599 > 80/99 | MPFOS | 4.407% | 0.013 |
| P1MHpS | Perfluoro-1-methylheptane sulfonate | 499 > 419 | MPFOS | 0.000% | 0.081 |
| P3MHpS | Perfluoro-3-methylheptane sulfonate | 499 > 130 | MPFOS | 93.305% | 0.043 |
| P3MHpA | Perfluoro-3-methylheptane acid | 413 > 169 | MPFOA | 0.000% | 0.101 |
| P4MHpS | Perfluoro-4-methylheptane sulfonate | 499 > 330 | MPFOS | 93.136% | 0.211 |
| P4MHpA | Perfluoro-4-methylheptane acid | 413 > 119 | MPFOA | 0.000% | 0.058 |
| P5MHpS | Perfluoro-5-methylheptane sulfonate | 499 > 130 | MPFOS | 96.864% | 0.120 |
| P5MHpA | Perfluoro-5-methylheptane acid | 413 > 219 | MPFOA | 0.000% | 0.025 |
| P6MHpS | Perfluoro-6-methylheptane sulfonate | 499 > 330 | MPFOS | 85.508% | 0.076 |
| P6MHpA | Perfluoro-6-methylheptane acid | 413 > 169 | MPFOA | 18.136 | 0.023 |
| P55DMHxS | Perfluoro-5,5-dimethylhexane sulfonate | 499 > 130 | MPFOS | 0.000% | 0.006 |
| P55DMHxA | Perfluoro-5,5-dimethylhexane acid | 413 > 219 | MPFOA | 0.000% | 0.017 |
| P44DMHxS | Perfluoro-4,4-dimethylhexane sulfonate | 499 > 330 | MPFOS | 85.678% | 0.006 |
| P44DMHxA | Perfluoro-4,4-dimethylhexane acid | 413 > 369 | MPFOA | 0.000% | 0.169 |
| P35DMHxS | Perfluoro-3,5-dimethylhexane sulfonate | 499 > 130 | MPFOS | 0.000% | 0.026 |
| P35DMHxA | Perfluoro-3,5-dimethylhexane acid | 413 > 369 | MPFOA | 0.000% | 0.173 |
| 4:2FTS | Sodium 1H,1H,2H,2H-perfluorohexane sulfonate (4:2) | 327 > 307/81 | M2-6:2FTS | 0.000% | 0.490 |
| 6:2FTS | Sodium 1H,1H,2H,2H-perfluorooctane sulfonate (6:2) | 427 > 407/81 | M2-6:2FTS | 0.000% | 0.549 |
| 8:2FTS | Sodium 1H,1H,2H,2H-perfluorodecane sulfonate (8:2) | 527 > 507/81 | M2-6:2FTS | 0.000% | 0.113 |
| 11CL-PF3OUdS | Potassium 11-chloroeicosafluoro-3-oxaundecane-1-sulfonate | 631 > 451/83 | MPFOS | 99.407% | 0.001 |
| 9CL-PF3ONS | Potassium 9-chlorohexadeca-fluoro-3-oxanonane-1-sulfonate | 531 > 351/99 | MPFOS | 100.000% | 0.007 |

**Table S2.** The spike recoveries (%) of six replicate samples and their relative standard deviation (RSD).

| PFAS | Sample 1 | Sample 2 | Sample 3 | Sample 4 | Sample 5 | Sample 6 | Average | RSD |
| --- | --- | --- | --- | --- | --- | --- | --- | --- |
| PFBA | 75.7 | 78.9 | 89.1 | 80.7 | 87.1 | 88.6 | 83.4 | 5.7 |
| PFPeA | 84.5 | 105.3 | 108.0 | 99.1 | 78.9 | 87.5 | 93.9 | 11.9 |
| PFHxA | 68.1 | 91.2 | 89.4 | 80.5 | 100.0 | 88.6 | 86.3 | 10.9 |
| PFHpA | 78.2 | 105.3 | 98.2 | 93.8 | 96.5 | 87.4 | 93.2 | 9.4 |
| PFDA | 58.2 | 61.5 | 67.5 | 69.3 | 78 | 74 | 68.1 | 7.4 |
| PFDoA | 91.2 | 119.5 | 65.8 | 116.8 | 78.9 | 84.3 | 92.7 | 21.4 |
| PFTeDA | 63.3 | 82.6 | 83.2 | 92.0 | 95.5 | 92.9 | 84.9 | 11.9 |
| PFTrDA | 89.4 | 97.5 | 78.9 | 88.6 | 87.1 | 94.3 | 89.3 | 6.4 |
| PFUdA | 76.4 | 100.0 | 102.7 | 93.8 | 79.9 | 86.4 | 89.9 | 10.7 |
| PFNA | 66.4 | 68.7 | 79.9 | 76.7 | 81.0 | 79.4 | 75.4 | 6.2 |
| PFOA | 64.8 | 63.5 | 71.2 | 70.5 | 74.4 | 74.2 | 69.8 | 4.6 |
| L-PFBS | 88.5 | 88.5 | 86.4 | 108.0 | 89.6 | 96.5 | 92.9 | 8.1 |
| L-PFHxS | 59.2 | 57.4 | 68.4 | 64.1 | 68.8 | 71.1 | 64.8 | 5.6 |
| L-PFHpS | 80.6 | 102.5 | 107.1 | 94.7 | 90.5 | 89.9 | 94.2 | 9.5 |
| L-PFDS | 80.0 | 88.9 | 99.1 | 86.4 | 78.6 | 89.6 | 87.1 | 7.4 |
| L-PFOS | 73.1 | 96.5 | 92.0 | 83.6 | 101.5 | 107.1 | 92.3 | 12.4 |
| 4:2FTS | 75.1 | 62.3 | 59.7 | 61.2 | 72.8 | 75.5 | 67.8 | 7.4 |
| 8:2FTS | 65.7 | 57.2 | 55.4 | 75.4 | 72.7 | 74.2 | 66.8 | 8.8 |
| 6:2FTS | 64.5 | 59.6 | 56.3 | 77.4 | 73.8 | 77.6 | 68.2 | 9.3 |
| 9CL-PF3ONS | 54.5 | 60.3 | 60.3 | 84.3 | 69.2 | 68.9 | 66.2 | 10.5 |
| 11CL-PF3OUdS | 63.7 | 63.3 | 66.1 | 86.5 | 70.8 | 68.8 | 69.9 | 8.7 |

**Table S3.** Crude and adjusted odds ratios (ORs) for the risk of breast cancer during the study period relative to the 17 PFASs by continuous and quartile in the logistic regression model.

| Exposure | | Range (ng/mL) | Cases/Controls | Crude OR (95%CI) | Adjusted OR (95%CI) | *P-*value |
| --- | --- | --- | --- | --- | --- | --- |
| PFOA |  | |  |  |  |  |
| Quartile 1 | <2.24 | | 96/161 | 1.0 | 1.0 | 0.000 |
| Quartile 2 | 2.24~3.35 | | 67/191 | 0.59 (0.40, 0.86)* | 0.66 (0.41, 1.08) |  |
| Quartile 3 | 3.35~5.11 | | 83/175 | 0.80 (0.55, 1.14) | 1.19 (0.75, 1.90) |  |
| Quartile 4 | ≥5.11 | | 127/130 | 1.64 (1.15, 2.33)* | 2.83 (1.79, 4.49)* |  |
| Ln (PFOA, ng/ml) ^a^ |  | |  | 1.27 (1.12, 1.45)* | 1.57 (1.31, 1.89)* |  |
| PFNA |  | |  |  |  |  |
| Quartile 1 | <1.11 | | 139/118 | 1.0 | 1.0 | 0.000 |
| Quartile 2 | 1.11~1.68 | | 78/180 | 0.37 (0.26, 0.53)* | 0.50 (0.32, 0.78)* |  |
| Quartile 3 | 1.68~2.32 | | 65/193 | 0.29 (0.20, 0.42)* | 0.37 (0.23, 0.59)* |  |
| Quartile 4 | ≥2.32 | | 91/166 | 0.47 (0.33, 0.66)* | 0.72 (0.46, 1.13) |  |
| Ln (PFNA, ng/ml) ^a^ |  | |  | 0.71 (0.63, 0.81)* | 0.84 (0.70, 1.01) |  |
| PFDA |  | |  |  |  |  |
| Quartile 1 | <0.62 | | 107/150 | 1.0 | 1.0 | 0.000 |
| Quartile 2 | 0.62~0.94 | | 66/192 | 0.48 (0.33, 0.70)* | 0.55 (0.34, 0.88)* |  |
| Quartile 3 | 0.94~1.44 | | 77/181 | 0.60 (0.41, 0.86)* | 0.72 (0.46, 1.14) |  |
| Quartile 4 | ≥1.44 | | 123/134 | 1.29 (0.91, 1.82) | 1.78 (1.14, 2.77)* |  |
| Ln (PFDA, ng/ml) ^a^ |  | |  | 1.09 (0.96, 1.24) | 1.27 (1.05, 1.52)* |  |
| PFUdA |  | |  |  |  |  |
| Quartile 1 | <0.50 | | 129/128 | 1.0 | 1.0 | 0.000 |
| Quartile 2 | 0.50~0.82 | | 104/154 | 0.67 (0.47, 0.95)* | 0.65 (0.42, 1.02) |  |
| Quartile 3 | 0.82~1.33 | | 99/159 | 0.62 (0.44, 0.88)* | 0.83 (0.54, 1.29) |  |
| Quartile 4 | ≥1.33 | | 41/216 | 0.19 (0.13, 0.29)* | 0.22 (0.13, 0.36)* |  |
| Ln (PFUdA, ng/ml) ^a^ |  | |  | 0.65 (0.56, 0.74)* | 0.71 (0.59, 0.84)* |  |
| PFDoA |  | |  |  |  |  |
| Quartile 1 | <0.05 | | 146/111 | 1.0 | 1.0 | 0.000 |
| Quartile 2 | 0.05~0.09 | | 98/160 | 0.47 (0.33, 0.66)* | 0.60 (0.39, 0.94)* |  |
| Quartile 3 | 0.09~0.14 | | 75/183 | 0.31 (0.22, 0.45)* | 0.50 (0.32, 0.77)* |  |
| Quartile 4 | ≥0.14 | | 54/203 | 0.20 (0.14, 0.30)* | 0.29 (0.18, 0.46)* |  |
| Ln (PFDoA, ng/ml) ^a^ |  | |  | 0.54 (0.47, 0.62)* | 0.65 (0.54, 0.77)* |  |
| PFTrDA |  | |  |  |  |  |
| Quartile 1 | <0.16 | | 230/27 | 1.0 | 1.0 | 0.000 |
| Quartile 2 | 0.16~0.38 | | 94/164 | 0.07 (0.04, 0.11)* | 0.06 (0.03, 0.10)* |  |
| Quartile 3 | 0.38~0.66 | | 31/227 | 0.02 (0.01, 0.03)* | 0.02 (0.01, 0.03)* |  |
| Quartile 4 | ≥0.66 | | 18/239 | 0.01 (0.01, 0.02)* | 0.01 (0.00, 0.02)* |  |
| Ln (PFTrDA, ng/ml) ^a^ |  | |  | 0.10 (0.08, 0.14)* | 0.10 (0.07, 0.14)* |  |
| PFTeDA |  | |  |  |  |  |
| Quartile 1 | <0.01 | | 209/48 | 1.0 | 1.0 | 0.000 |
| Quartile 2 | 0.01~0.04 | | 74/184 | 0.09 (0.06, 0.14)* | 0.05 (0.03, 0.09)* |  |
| Quartile 3 | 0.04~0.07 | | 52/206 | 0.06 (0.04, 0.09)* | 0.04 (0.02, 0.07)* |  |
| Quartile 4 | ≥0.07 | | 38/219 | 0.04 (0.03, 0.06)* | 0.03 (0.02, 0.06)* |  |
| Ln (PFTeDA, ng/ml) ^a^ |  | |  | 0.26 (0.22, 0.31)* | 0.23 (0.18, 0.30)* |  |
| PFHxS |  | |  |  |  |  |
| Quartile 1 | <0.70 | | 128/129 | 1.0 | 1.0 | 0.000 |
| Quartile 2 | 0.70~1.45 | | 87/171 | 0.51 (0.36, 0.73)* | 0.50 (0.32, 0.78)* |  |
| Quartile 3 | 1.45~2.40 | | 81/177 | 0.46 (0.32, 0.66)* | 0.46 (0.29, 0.72)* |  |
| Quartile 4 | ≥2.40 | | 77/180 | 0.43 (0.30, 0.62)* | 0.44 (0.28, 0.68)* |  |
| Ln (PFHxS, ng/ml) ^a^ |  | |  | 0.69 (0.60, 0.78)* | 0.73 (0.62, 0.87)* |  |
| PFHpS |  | |  |  |  |  |
| Quartile 1 | <0.26 | | 106/151 | 1.0 | 1.0 | 0.049 |
| Quartile 2 | 0.26~0.52 | | 96/162 | 0.84 (0.59, 1.20) | 0.74 (0.48, 1.15) |  |
| Quartile 3 | 0.52~0.89 | | 95/163 | 0.83 (0.58, 1.18) | 0.78 (0.50, 1.22) |  |
| Quartile 4 | ≥0.89 | | 76/181 | 0.60 (0.42, 0.86)* | 0.63 (0.39, 1.00)* |  |
| Ln (PFHpS, ng/ml) ^a^ |  | |  | 0.80 (0.71, 0.91)* | 0.78 (0.65, 0.93)* |  |
| PFOS |  | |  |  |  |  |
| Quartile 1 | <7.45 | | 119/138 | 1.0 | 1.0 |  |
| Quartile 2 | 7.45~12.18 | | 85/173 | 0.57 (0.40 0.81)* | 0.61 (0.40, 0.95)* | 0.002 |
| Quartile 3 | 12.18~17.72 | | 83/175 | 0.55 (0.38, 0.79)* | 0.58 (0.37, 0.91)* |  |
| Quartile 4 | ≥17.72 | | 86/171 | 0.58 (0.41, 0.83)* | 0.64 (0.41, 1.00) |  |
| Ln (PFOS, ng/ml) ^a^ |  | |  | 0.79 (0.70, 0.90)* | 0.81 (0.68, 0.96)* |  |
| P3MHpS |  | |  |  |  |  |
| Quartile 1 | <0.46 | | 96/161 | 1.0 | 1.0 | 0.945 |
| Quartile 2 | 0.46~0.86 | | 92/166 | 0.93 (0.65, 1.33) | 0.87 (0.55, 1.37) |  |
| Quartile 3 | 0.86~1.54 | | 95/163 | 0.98 (0.68, 1.40) | 0.89 (0.57, 1.39) |  |
| Quartile 4 | ≥1.54 | | 90/167 | 0.90 (0.63, 1.30) | 0.87 (0.55, 1.37) |  |
| Ln (P3MHpS, ng/ml) ^a^ |  | |  | 0.96 (0.84, 1.09) | 0.89 (0.75, 1.06) |  |
| P4MHpS |  | |  |  |  |  |
| Quartile 1 | <0.59 | | 99/158 | 1.0 | 1.0 | 0.664 |
| Quartile 2 | 0.59~1.18 | | 96/162 | 0.95 (0.66, 1.35) | 0.87 (0.55, 1.36) |  |
| Quartile 3 | 1.18~2.08 | | 92/166 | 0.89 (0.62, 1.27) | 0.89 (0.57, 1.40) |  |
| Quartile 4 | ≥2.08 | | 86/171 | 0.80 (0.56, 1.15) | 0.65 (0.41, 1.02) |  |
| Ln (P4MHpS, ng/ml) ^a^ |  | |  | 0.92 (0.81, 1.04) | 0.86 (0.73, 1.03) |  |
| P5MHpS |  | |  |  |  |  |
| Quartile 1 | <0.90 | | 101/156 | 1.0 | 1.0 | 0.366 |
| Quartile 2 | 0.90~1.82 | | 88/170 | 0.80 (0.56, 1.15) | 0.81 (0.52, 1.27) |  |
| Quartile 3 | 1.82~3.10 | | 99/159 | 0.96 (0.68, 1.37) | 0.91 (0.58, 1.42) |  |
| Quartile 4 | ≥3.10 | | 85/172 | 0.76 (0.53, 1.10) | 0.66 (0.42, 1.03) |  |
| Ln (P5MHpS, ng/ml) ^a^ |  | |  | 0.91 (0.80, 1.03) | 0.85 (0.72, 1.01) |  |
| P6MHpS |  | |  |  |  |  |
| Quartile 1 | <0.81 | | 97/160 | 1.0 | 1.0 | 0.472 |
| Quartile 2 | 0.81~1.73 | | 101/157 | 1.06 (0.74, 1.51) | 0.78 (0.50, 1.22) |  |
| Quartile 3 | 1.73~2.96 | | 90/168 | 0.88 (0.62, 1.27) | 0.85 (0.55, 1.33) |  |
| Quartile 4 | ≥2.96 | | 85/172 | 0.82 (0.57, 1.17) | 0.79 (0.50, 1.24) |  |
| Ln (P6MHpS, ng/ml) ^a^ |  | |  | 0.99 (0.87, 1.12) | 1.00 (0.84, 1.18) |  |
| P45DMHxS |  | |  |  |  |  |
| Quartile 1 | <0.03 | | 102/155 | 1.0 | 1.0 |  |
| Quartile 2 | 0.03~0.08 | | 93/165 | 0.86 (0.60, 1.22) | 0.62 (0.40, 0.98)* |  |
| Quartile 3 | 0.08~0.15 | | 92/166 | 0.84 (0.59, 1.20) | 0.84 (0.54, 1.31) | 0.527 |
| Quartile 4 | ≥0.15 | | 86/171 | 0.76 (0.53, 1.10) | 0.61 (0.39, 0.95)* |  |
| Ln (P45DMHxS, ng/ml) ^a^ |  | |  | 0.93 (0.82, 1.05) | 0.88 (0.74, 1.05) |  |
| 11CL-PF3OUdS |  | |  |  |  |  |
| Quartile 1 | <0.06 | | 96/161 | 1.0 | 1.0 | 0.977 |
| Quartile 2 | 0.06~0.11 | | 93/165 | 0.95 (0.66, 1.35) | 0.66 (0.42, 1.04) |  |
| Quartile 3 | 0.11~0.21 | | 92/166 | 0.93 (0.65, 1.33) | 0.74 (0.47, 1.15) |  |
| Quartile 4 | ≥0.21 | | 92/165 | 0.94 (0.65, 1.34) | 0.70 (0.45, 1.10) |  |
| Ln (11CL-PF3OUdS, ng/ml) ^a^ |  | |  | 0.91 (0.80, 1.03) | 0.83 (0.69, 0.99)* |  |
| 9CL-PF3ONS |  | |  |  |  |  |
| Quartile 1 | <4.69 | | 97/160 | 1.0 | 1.0 | 0.756 |
| Quartile 2 | 4.69~9.15 | | 97/161 | 0.99 (0.70, 1.42) | 0.77 (0.49, 1.20) |  |
| Quartile 3 | 9.15~15.11 | | 87/171 | 0.84 (0.59, 1.20) | 0.67 (0.43, 1.05) |  |
| Quartile 4 | ≥15.11 | | 92/165 | 0.92 (0.64, 1.32) | 0.70 (0.45, 1.10) |  |
| Ln (9CL-PF3ONS, ng/ml) ^a^ |  | |  | 0.94 (0.83, 1.07) | 0.85 (0.71, 1.01) |  |

Abbreviations: OR, odds ratios; CI, confidence interval; PFOA, perfluorooctanoic acid; PFNA, perfluoro-n-nonanoic acid; PFDA, perfluoro-n-decanoic acid; PFUdA, perfluoro-n-undecanoic acid; PFDoA, perfluoro-n-dodecanoic acid; PFTrDA, perfluoro-n-tridecanoic acid; PFTeDA, perfluoro-n-tetradecanoic acid; PFHxS, perfluorohexane sulfonate; PFHpS, perfluoroheptane sulfonate; PFOS, perfluorooctanesulfonic acid; P3MHpS, perfluoro-3-methylheptane sulfonate; P4MHpS, perfluoro-4-methylheptane sulfonate; P5MHpS, perfluoro-5-methylheptane sulfonate; P6MHpS, perfluoro-6-methylheptane sulfonate; P45DMHxS, perfluoro-4,5-dimethylhexane sulfonate; 11CL-PF3OUdS, potassium 11-chloroeicosafluoro-3-oxaundecane-1-sulfonate; 9CL-PF3ONS, potassium 9-chlorohexadeca-fluoro-3-oxanonane-1-sulfonate.

Note: Final multivariable models adjusted for age at baseline (years), BMI (kg/m2), smoking history, age at menarche (years), age of menopause (years), parity, breastfeeding duration (months), use of estrogen or estrogen replacement therapy, family history of breast cancer, education (years), monthly household income per capita (RMB/month), red meat consumption, pickled, fried, smoked, and barbecued food consumption.

^a^ The OR and 95% CI of breast cancer were estimated by one SD increase in ln-transformed PFASs as continuous variables.

* *p*-value for trend < 0.05.

**Table S4.** The variable posterior inclusion probabilities (PIPs) estimated by Bayesian kernel machine regression (BKMR) model of the 17 PFASs.

| Number | Variable | PIP |
| --- | --- | --- |
| 1 | PFOA | 1.0000 |
| 2 | PFNA | 0.0000 |
| 3 | PFDA | 1.0000 |
| 4 | PFUdA | 1.0000 |
| 5 | PFDoA | 0.2636 |
| 6 | PFTrDA | 1.0000 |
| 7 | PFTeDA | 0.3768 |
| 8 | PFHxS | 0.0000 |
| 9 | PFHpS | 0.0974 |
| 10 | PFOS | 0.0000 |
| 11 | P3MHpS | 0.0000 |
| 12 | P4MHpS | 0.0000 |
| 13 | P5MHpS | 0.0000 |
| 14 | P6MHpS | 0.0000 |
| 15 | P45DMHxS | 0.1332 |
| 16 | 11CL-PF3OUdS | 0.1634 |
| 17 | 9CL-PF3ONS | 0.0000 |

Abbreviations: PIPs, posterior inclusion probabilities; BKMR, Bayesian kernel machine regression; PFOA, perfluorooctanoic acid; PFNA, perfluoro-n-nonanoic acid; PFDA, perfluoro-n-decanoic acid; PFUdA, perfluoro-n-undecanoic acid; PFDoA, perfluoro-n-dodecanoic acid; PFTrDA, perfluoro-n-tridecanoic acid; PFTeDA, perfluoro-n-tetradecanoic acid; PFHxS, perfluorohexane sulfonate; PFHpS, perfluoroheptane sulfonate; PFOS, perfluorooctanesulfonic acid; P3MHpS, perfluoro-3-methylheptane sulfonate; P4MHpS, perfluoro-4-methylheptane sulfonate; P5MHpS, perfluoro-5-methylheptane sulfonate; P6MHpS, perfluoro-6-methylheptane sulfonate; P45DMHxS, perfluoro-4,5-dimethylhexane sulfonate; 11CL-PF3OUdS, potassium 11-chloroeicosafluoro-3-oxaundecane-1-sulfonate; 9CL-PF3ONS, potassium 9-chlorohexadeca-fluoro-3-oxanonane-1-sulfonate.

**Table S5.** Crude and adjusted odds ratios (ORs) for the risk of breast cancer relative to the natural logarithm transformed concentrations of three selected perfluoroalkyl substances (PFASs) stratified by parity in the logistic regression model.

| (ng/ml) | Parity | Cases/controls | Crude OR (95% CI) | Cases/controls | Adjusted OR (95% CI) |
| --- | --- | --- | --- | --- | --- |
| PFOA |  |  |  |  |  |
|  | < 2 | 246/553 | 2.85 (2.12, 3.82)* | 212/390 | 4.62 (2.91, 7.34)* |
|  | ≥ 2 | 126/95 | 1.45 (0.91, 2.30) | 103/57 | 1.39 (0.64, 3.03) |
| PFDA |  |  |  |  |  |
|  | < 2 | 246/553 | 1.63 (1.21, 2.19)* | 212/390 | 1.92 (1.28, 2.90)* |
|  | ≥ 2 | 126/95 | 4.61 (2.39, 8.87)* | 103/57 | 6.40 (1.99, 20.57)* |
| PFTrDA |  |  |  |  |  |
|  | < 2 | 246/553 | 0.04 (0.03, 0.07)* | 212/390 | 0.03 (0.01, 0.05)* |
|  | ≥ 2 | 126/95 | 0.02 (0.01, 0.05)* | 103/57 | 0.01 (0.00, 0.08)* |

Abbreviations: OR, odds ratio; CI, confidence interval; PFOA, perfluoro-n-octanoic acid; PFDA, perfluoro-n-decanoic acid; PFTrDA, perfluoro-n-tridecanoic acid.

Note: Final multivariable model were adjusted for age at baseline (years), BMI (kg/m^2^), smoking history, age at menarche (years), age of menopause (years), parity, breastfeeding duration (months), use of estrogen or estrogen replacement therapy, family history of breast cancer, education (years), monthly household income per capita (RMB/month), red meat consumption, pickled, fried, smoked, and barbecued food consumption. The OR and 95% CI of breast cancer were estimated by one SD increase in ln-transformed PFOA, PFDA, and PFTrDA as continuous variables.

* *p*-value for trend < 0.05.

**Table S6.** Crude and adjusted odds ratios (ORs) for the risk of breast cancer relative to the natural logarithm transformed concentrations of three selected perfluoroalkyl substances (PFASs) stratified by breastfeeding duration in the logistic regression model.

| (ng/ml) | Breastfeeding duration (months) | Cases/controls | Crude OR (95% CI) | Cases/controls | Adjusted OR  (95% CI) |
| --- | --- | --- | --- | --- | --- |
| PFOA |  |  |  |  |  |
|  | < 13 | 139/422 | 2.65 (1.87, 3.76)* | 128/295 | 4.00 (2.36, 6.77)* |
|  | ≥ 13 | 211/210 | 2.23 (1.52, 3.27)* | 187/152 | 3.10 (1.76, 5.46)* |
| PFDA |  |  |  |  |  |
|  | < 13 | 139/422 | 1.35 (0.96, 1.90) | 128/295 | 1.72 (1.11, 2.67)* |
|  | ≥ 13 | 211/210 | 3.07 (1.99, 4.74)* | 187/152 | 4.89 (2.38, 10.04)* |
| PFTrDA |  |  |  |  |  |
|  | < 13 | 139/422 | 0.06 (0.03, 0.10)* | 128/295 | 0.04 (0.02, 0.08)* |
|  | ≥ 13 | 211/210 | 0.03 (0.01, 0.05)* | 187/152 | 0.02 (0.01, 0.05)* |

Abbreviations: OR, odds ratio; CI, confidence interval; PFOA, perfluoro-n-octanoic acid; PFDA, perfluoro-n-decanoic acid; PFTrDA, perfluoro-n-tridecanoic acid.

Note: Final multivariable model were adjusted for age at baseline (years), BMI (kg/m^2^), smoking history, age at menarche (years), age of menopause (years), parity, breastfeeding duration (months), use of estrogen or estrogen replacement therapy, family history of breast cancer, education (years), monthly household income per capita (RMB/month), red meat consumption, pickled, fried, smoked, and barbecued food consumption. The OR and 95% CI of breast cancer were estimated by one SD increase in ln-transformed PFOA, PFDA, and PFTrDA as continuous variables.

* *p*-value for trend < 0.05.

***Part 4***

***Supplemental Figures (Figure S1- Figure S5)***


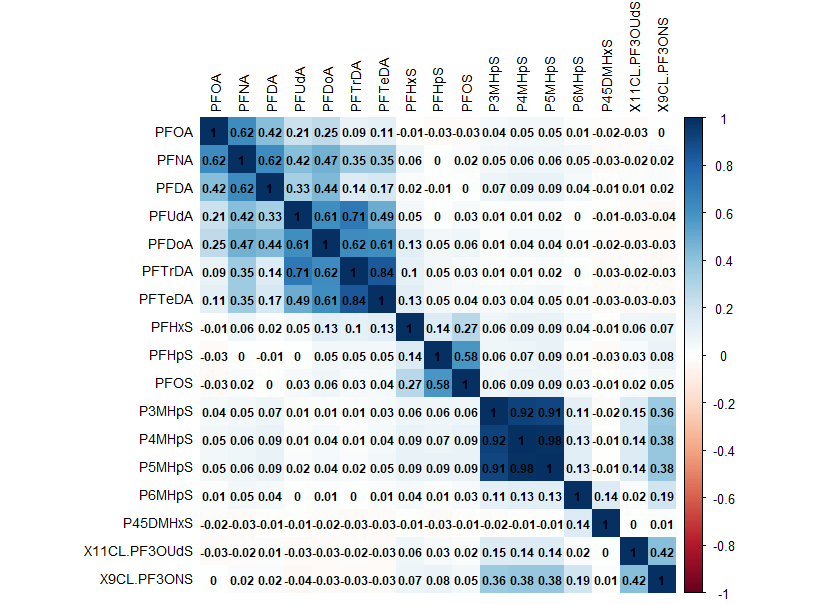


**Figure S1.** Spearman’s correlation coefficients for the plasma concentrations of perfluoroalkyl substances (PFASs) measured in the cases and the controls. The concentrations of PFASs were quantified on an ultra-performance liquid chromatography (UPLC) system coupled to a 5500 Q-Trap triple quadrupole mass spectrometry.

Abbreviations: PFOA, perfluorooctanoic acid; PFNA, perfluoro-n-nonanoic acid; PFDA, perfluoro-n-decanoic acid; PFUdA, perfluoro-n-undecanoic acid; PFDoA, perfluoro-n-dodecanoic acid; PFTrDA, perfluoro-n-tridecanoic acid; PFTeDA, perfluoro-n-tetradecanoic acid; PFHxS, perfluorohexane sulfonate; PFHpS, perfluoroheptane sulfonate; PFOS, perfluorooctanesulfonic acid; P3MHpS, perfluoro-3-methylheptane sulfonate; P4MHpS, perfluoro-4-methylheptane sulfonate; P5MHpS, perfluoro-5-methylheptane sulfonate; P6MHpS, perfluoro-6-methylheptane sulfonate; P45DMHxS, perfluoro-4,5-dimethylhexane sulfonate; 11CL-PF3OUdS, potassium 11-chloroeicosafluoro-3-oxaundecane-1-sulfonate; 9CL-PF3ONS, potassium 9-chlorohexadeca-fluoro-3-oxanonane-1-sulfonate.


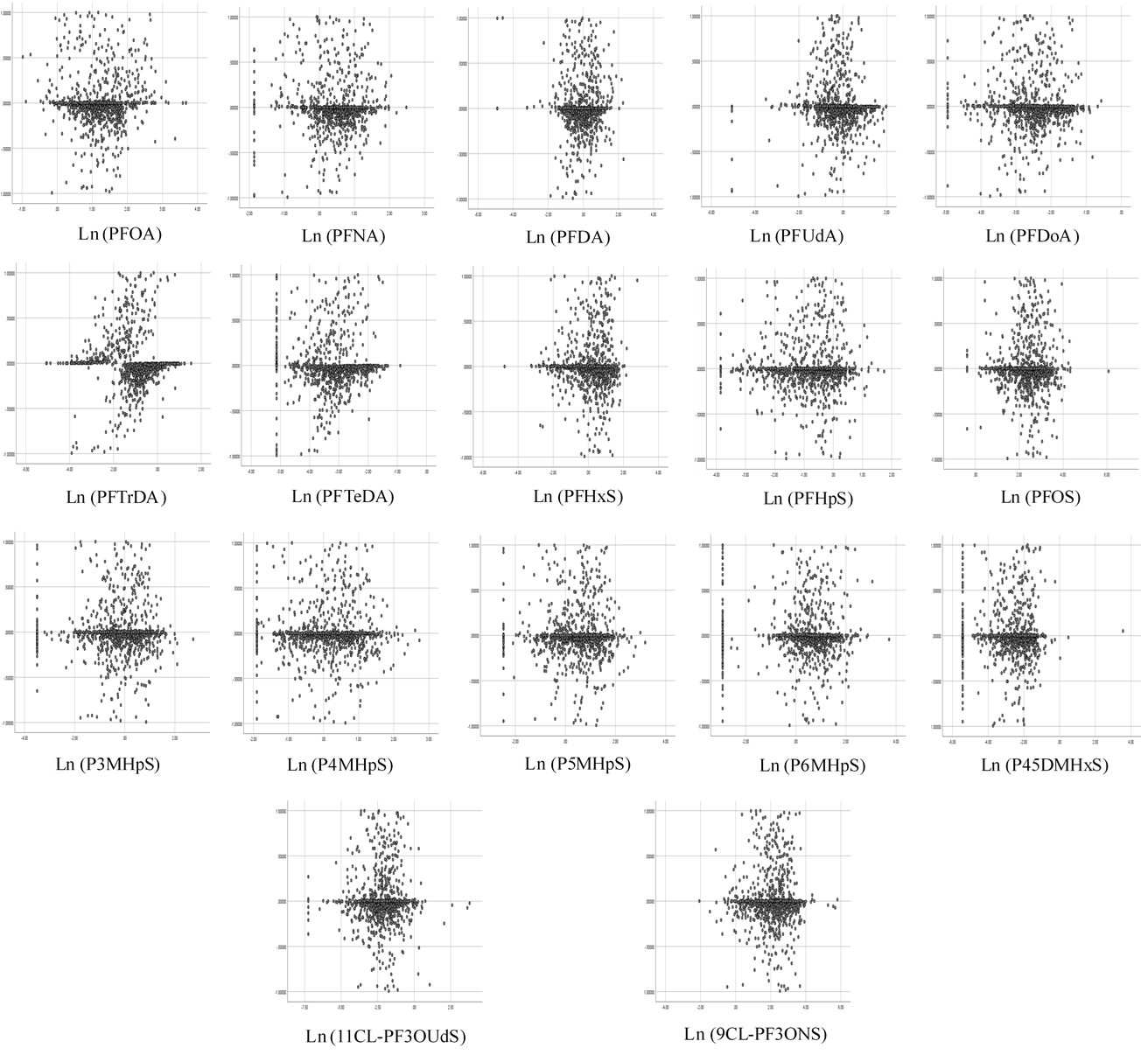


**Figure S2.** Scatter plots of residual distributions of the17 Ln (PFASs).

Abbreviations: PFOA, perfluorooctanoic acid; PFNA, perfluoro-n-nonanoic acid; PFDA, perfluoro-n-decanoic acid; PFUdA, perfluoro-n-undecanoic acid; PFDoA, perfluoro-n-dodecanoic acid; PFTrDA, perfluoro-n-tridecanoic acid; PFTeDA, perfluoro-n-tetradecanoic acid; PFHxS, perfluorohexane sulfonate; PFHpS, perfluoroheptane sulfonate; PFOS, perfluorooctanesulfonic acid; P3MHpS, perfluoro-3-methylheptane sulfonate; P4MHpS, perfluoro-4-methylheptane sulfonate; P5MHpS, perfluoro-5-methylheptane sulfonate; P6MHpS, perfluoro-6-methylheptane sulfonate; P45DMHxS, perfluoro-4,5-dimethylhexane sulfonate; 11CL-PF3OUdS, potassium 11-chloroeicosafluoro-3-oxaundecane-1-sulfonate; 9CL-PF3ONS, potassium 9-chlorohexadeca-fluoro-3-oxanonane-1-sulfonate.


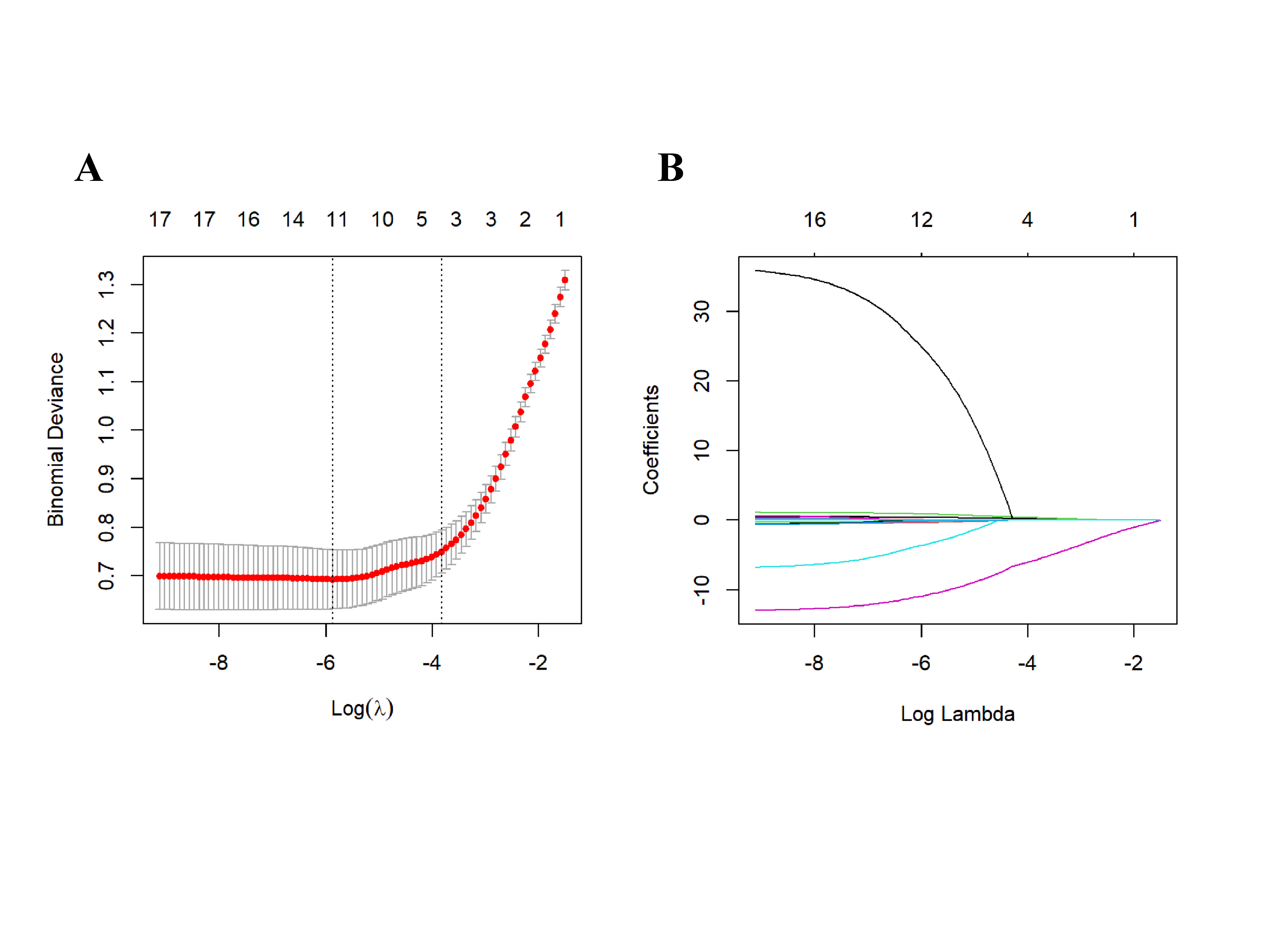


**Figure S3.** PFASs selection using the least absolute shrinkage and selection operator (LASSO) regression model. (**A**) LASSO coefficient of the 17 PFASs in breast cancer; (**B**) the optimal penalty coefficient (λ = 0.0218) in the LASSO regression identified with the minimum criterion.

**
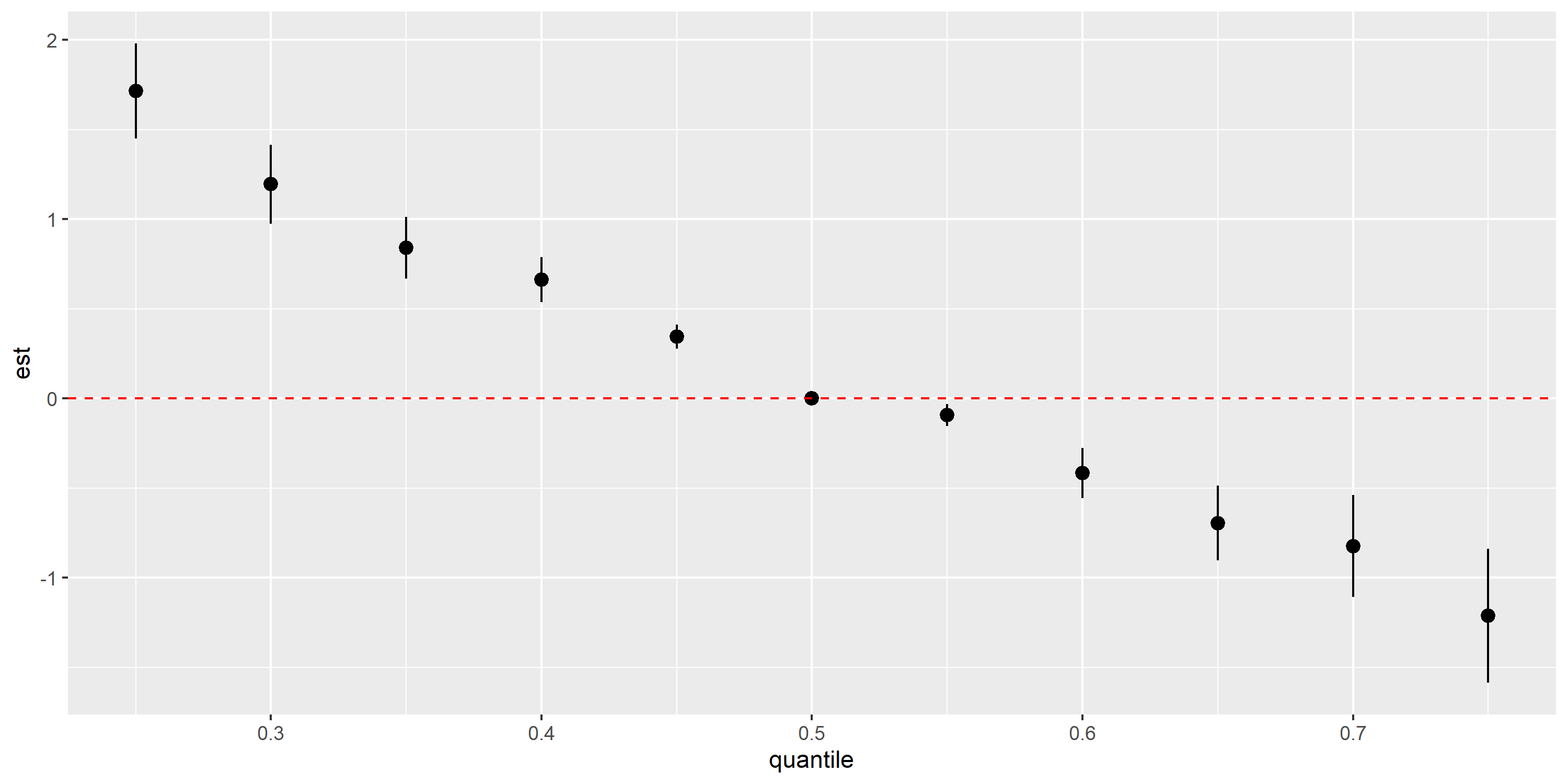
**

**A**

**B**


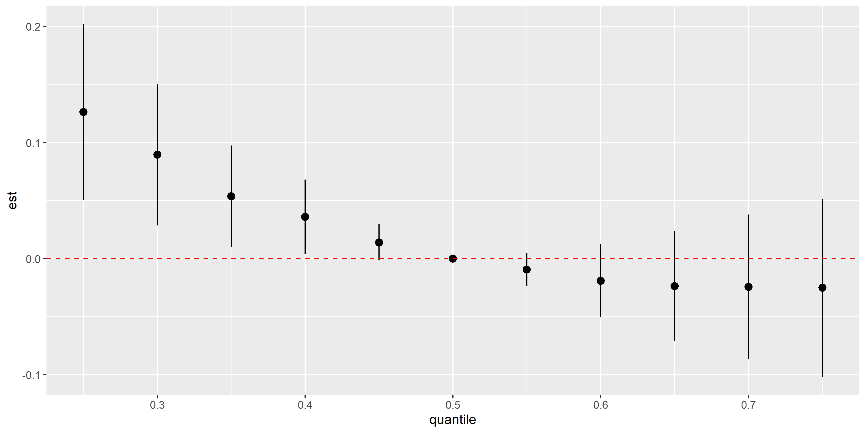


**C**


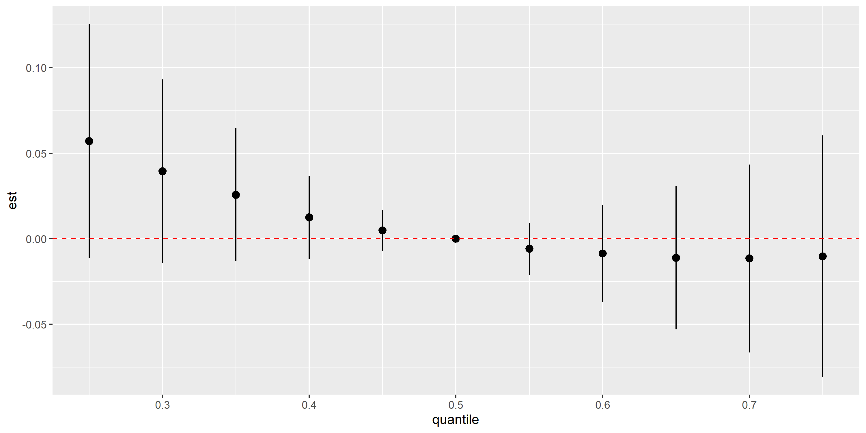


**Figure S4**. Joint effect estimates with 95% CI for the breast cancer in relation to PFCAs (**A**) PFSAs (**B**) and PFSA isomers (**C**) in the Bayesian kernel machine regression (BKMR) model, adjusted for age at baseline (years), BMI (kg/m^2^), smoking history, age at menarche (years), age of menopause (years), parity, breastfeeding duration (months), use of estrogen or estrogen replacement therapy, family history of breast cancer, education (years), monthly household income per capita (RMB/month), red meat consumption, pickled, fried, smoked, and barbecued food consumption.

Abbreviations: CI, confidence interval; BKMR, Bayesian kernel machine regression; BMI, body mass index.

Note: All the chemicals at particular percentiles (from 0.25 to 0.75 increment by 0.05) were compared to all the chemicals at their 50th percentile. The ∑PFCAs included PFOA, PFNA, PFDA, PFUdA, PFDoA, PFTrDA, and PFTeDA. The ∑PFSAs included PFHxS, PFHpS, and PFOS. The ∑PFSA isomers included P3MHpS, P4MHpS, P5MHpS, P6MHpS, P45DMHxS, 11CL-PF3OUdS, and 9CL-PF3ONS.

**A**


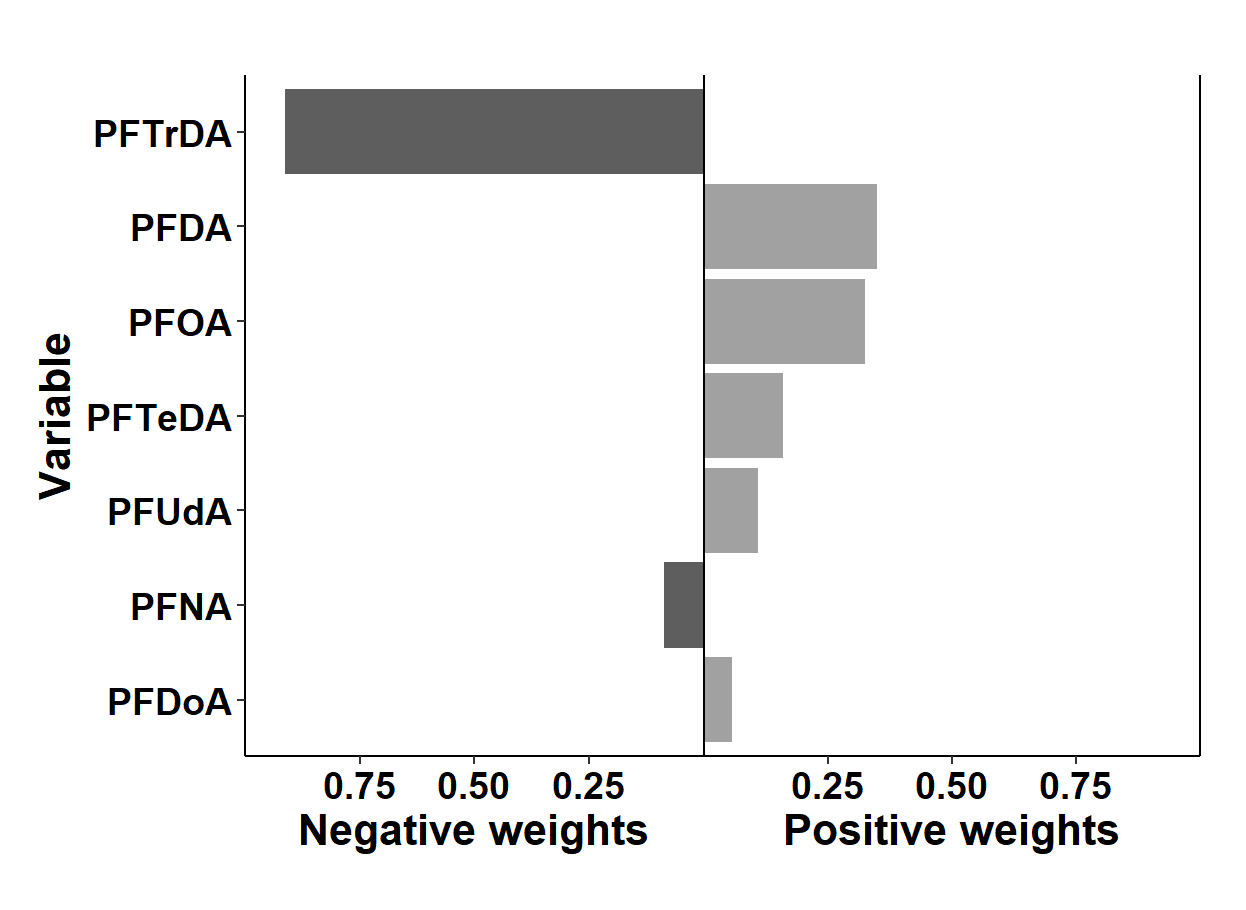

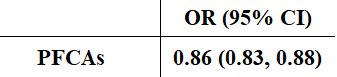

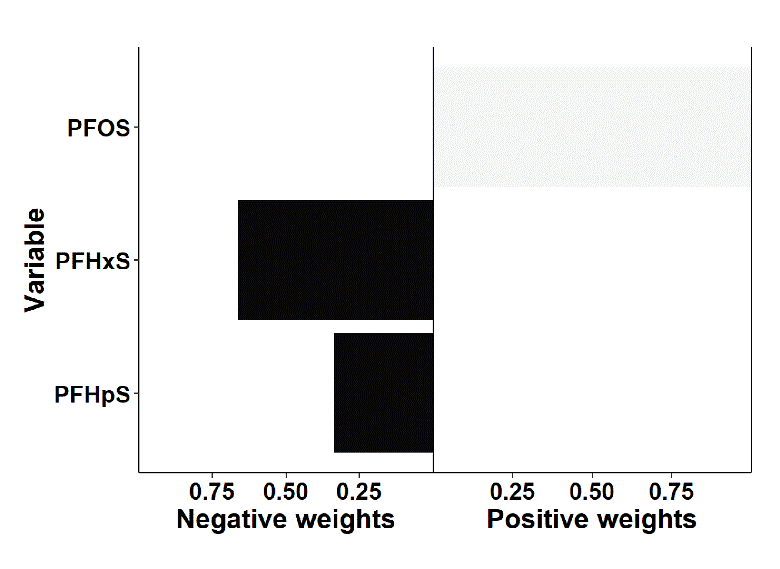

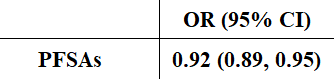


**B**

**C**


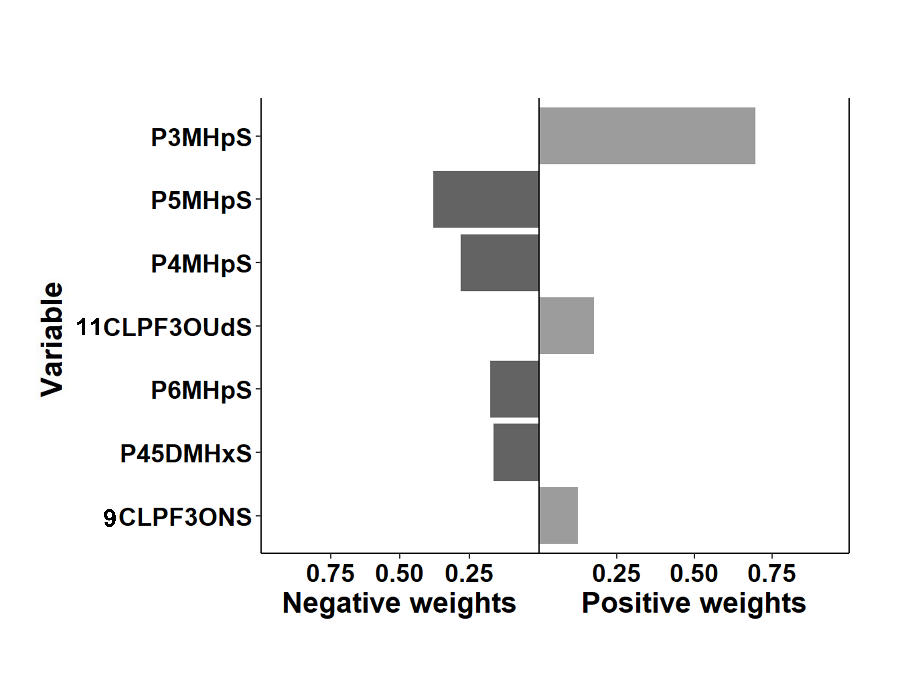

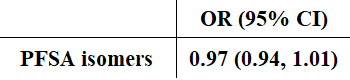


**Figure S5.** Mixture effects of PFCAs (**A**) PFSAs (**B**) and PFSA isomers (**C**) on breast cancer in the quantile g-computation model, adjusted for age at baseline (years), BMI (kg/m^2^), smoking history, age at menarche (years), age of menopause (years), parity, breastfeeding duration (months), use of estrogen or estrogen replacement therapy, family history of breast cancer, education (years), monthly household income per capita (RMB/month), red meat consumption, pickled, fried, smoked, and barbecued food consumption.

Abbreviations: PFCA, perfluorinated carboxylic acid; PFOA, perfluorooctanoic acid; PFNA, perfluoro-n-nonanoic acid; PFDA, perfluoro-n-decanoic acid; PFUdA, perfluoro-n-undecanoic acid; PFDoA, perfluoro-n-dodecanoic acid; PFTrDA, perfluoro-n-tridecanoic acid; PFTeDA, perfluoro-n-tetradecanoic acid; PFSA, perfluorinated sulfonic acid; PFHxS, perfluorohexane sulfonate; PFHpS, perfluoroheptane sulfonate; PFOS, perfluorooctanesulfonic acid; P3MHpS, perfluoro-3-methylheptane sulfonate; P4MHpS, perfluoro-4-methylheptane sulfonate; P5MHpS, perfluoro-5-methylheptane sulfonate; P6MHpS, perfluoro-6-methylheptane sulfonate; P45DMHxS, perfluoro-4,5-dimethylhexane sulfonate; 11CL-PF3OUdS, potassium 11-chloroeicosafluoro-3-oxaundecane-1-sulfonate; 9CL-PF3ONS, potassium 9-chlorohexadeca-fluoro-3-oxanonane-1-sulfonate; OR, odds ratio; CI, confidence interval; BMI, body mass index.

Note: The weight of each PFAS represents the proportion of directional (positive/negative) effect that is accounted for by each PFAS. The ∑PFCAs included PFOA, PFNA, PFDA, PFUdA, PFDoA, PFTrDA, and PFTeDA. The ∑PFSAs included PFHxS, PFHpS, and PFOS. The ∑PFSA isomers included P3MHpS, P4MHpS, P5MHpS, P6MHpS, P45DMHxS, 11CL-PF3OUdS, and 9CL-PF3ONS.
